# Human virome profiling identified CMV as the major viral driver of a high accumulation of senescent CD8^+^ T cells in patients with advanced NSCLC

**DOI:** 10.1126/sciadv.adh0708

**Published:** 2023-11-08

**Authors:** Marie Naigeon, Matthieu Roulleaux Dugage, François-Xavier Danlos, Lisa Boselli, Jean-Mehdi Jouniaux, Caroline de Oliveira, Roberto Ferrara, Boris Duchemann, Caroline Berthot, Lou Girard, Ronan Flippot, Laurence Albiges, Siham Farhane, Patrick Saulnier, Ludovic Lacroix, Frank Griscelli, Gabriel Roman, Tyler Hulett, Aurélien Marabelle, Lydie Cassard, Benjamin Besse, Nathalie Chaput

**Affiliations:** ^1^ Laboratoire d'Immunomonitoring en Oncologie, INSERM US23, CNRS UMS 3655, Gustave Roussy, Villejuif, France.; ^2^ Faculté de Médecine, Université Paris-Saclay, Le Kremlin-Bicêtre, France.; ^3^ Faculté de Pharmacie, Université Paris-Saclay, Orsay, France.; ^4^ Service d’Oncologie Médicale, Hôpital Européen Georges Pompidou, AP-HP, Paris, France.; ^5^ Département d’Innovation Thérapeutique et d’Essais Précoces (DITEP), Gustave Roussy, Villejuif, France.; ^6^ Laboratoire de Recherche Translationnelle en Immunothérapie (LRTI), INSERM U1015 and Centre d’Investigation Clinique BIOTHERIS, INSERM CIC1428, Gustave Roussy, Villejuif, France.; ^7^ Università Vita-Salute San Raffaele, Milan, Italy.; ^8^ Département d’oncologie thoracique et médicale, Hôpitaux Universitaires Paris Seine-Saint-Denis, Hôpital Avicenne, AP-HP, Bobigny, France.; ^9^ Département de Médecine Oncologique, Gustave Roussy, Villejuif, France.; ^10^ AMMICa, UAR 3655/US23, Gustave Roussy, Villejuif, France.; ^11^ Département de Biologie Médicale et Pathologie Médicales, Gustave Roussy, Villejuif, France.; ^12^ CDI Laboratories Inc., 1 N. Haven Street, Suite B001, Baltimore, MD 21224, USA.

## Abstract

Circulating senescent CD8^+^ T (T_8_sen) cells are characterized by a lack of proliferative capacities but retain cytotoxic activity and have been associated to resistance to immunotherapy in patients with advanced non–small cell lung cancer (aNSCLC). We aimed to better characterize T_8_sen and to determine which factors were associated with their accumulation in patients with aNSCLC. Circulating T_8_sen cells were characterized by a higher expression of SA-βgal and the transcription factor T-bet, confirming their senescent status. Using whole virome profiling, cytomegalovirus (CMV) was the only virus associated with T_8_sen. CMV was necessary but not sufficient to explain high accumulation of T_8_sen (T_8_sen^high^ status). In CMV^+^ patients, the proportion of T_8_sen cells increased with cancer progression. Last, CMV-induced T_8_sen^high^ phenotype but not CMV seropositivity itself was associated with worse progression-free and overall survival in patients treated with anti–PD-(L)1 therapy but not with chemotherapy. Overall, CMV is the unique viral driver of T_8_sen-driven resistance to anti–PD-(L)1 antibodies in patients with aNSCLC.

## Teaser

CMV is the main viral driver of T_8_sen-induced resistance to anti-PD-(L)1 in patients with aNSCLC.

## INTRODUCTION

Immune escape and protumoral inflammation have been identified as processes contributing to tumor growth (*1*). While cytotoxic CD8^+^ T cells play a key role in the control of cancer by recognizing tumor-specific antigens bound to type I major histocompatibility complex (MHC I), several mechanisms can hamper their function. Among those, the characterization of the programmed cell death protein 1 (PD-1)/programmed death-ligand 1/2 (PD-L1/2) axis (*2*) has led to the development of therapeutic anti–PD-1 and anti–PD-L1 antibodies [immune checkpoint blockers (ICBs)]. Patients with advanced non–small cell lung cancer (aNSCLC) yield great benefit from such approaches, but not all patients respond to treatment [~40% as a monotherapy in patients with high PD-L1 expression (*3*) and 50 to 60% when combined with conventional chemotherapy (*4*, *5*)]. Biomarkers of response exist at the tumor level (PD-L1 expression by tumor cells or tumor-infiltrating cells, a high tumor mutational burden, and microsatellite instability-high (MSI-H) or Mismatch Repair-deficiency (dMMR) status), but there is a lack of circulating biomarkers easily usable in the clinical practice (*6*). Therefore, new biomarkers are highly needed to better characterize the host immune system guide clinical choices and predict ICB response.

Aging is associated with several structural and functional changes in the immune system, which are classified under the term “immunosenescence,” affecting both innate and adaptive response. Along with the persistence of proinflammatory stimuli, immunosenescence is also associated with a senescent-associated secretory phenotype (SASP) of immune cells (*7*), which contributes to “inflammaging,” i.e., a low level of systemic inflammation with higher levels of interleukin-6 (IL-6), IL-8, and C-reactive protein (*8*).

Persistent antigenic stimulation induced by cancer (*9*), chronic viral infections (*10*, *11*), chronic inflammatory diseases (*12*), and DNA damage caused by oxidative stress (*13*) or chemotherapy (*14*, *15*) may also induce senescence in both innate and adaptive immune systems, underlying the fact that immunosenescence is a complex multifactorial phenomenon. In our previous study on a cohort of ICB-treated patients with aNSCLC, we described CD28^−^CD57^+^KLRG1^+^CD8^+^ T cells. These cells were poorly proliferative, produced high levels of proinflammatory cytokines and low levels of IL-2 that are functional characteristics of senescent T cells. We have previously identified a cut-off value of 39.5%; patients with more than 39.5% of circulating CD28^−^CD57^+^KLRG1^+^CD8^+^ T cells (T_8_sen^high^) were resistant to ICB (*16*). However, the factors associated with T cell senescence in patients with cancer remain poorly understood. In this work, we aimed to better characterize CD28^−^CD57^+^KLRG1^+^CD8^+^ T (T_8_sen) cells and to investigate several etiologies of premature immune aging, including cancer, systemic inflammation, and viral infections.

## RESULTS

### Characteristics of patients with aNSCLC

Overall, the clinical and biological data from 238 patients with aNSCLC are analyzed in this study, of which 61 received polychemotherapy treatment [Identification of Marker of Primary or Acquired Resistance to Anti Tumorous Treatment (MSN) study, PCT-treated], 177 were treated by ICB monotherapy [*n* = 121 in PREMIS (Predictive Markers of Immune-related Adverse Events in Patients Treated with Immune Stimulatory Drugs) study and *n* = 56 in Monitoring of Circulating Tumor Cells (CTC) study]. Median age across all cohorts included in the clinical analysis was 63.35 (31.50 to 92.86) years old. Patients had a median of 2 (0 to 7) metastatic sites. Cytomegalovirus (CMV) serology data were available for 212 patients (42 PCT-treated and 170 ICB-treated) of which 125 (58.96%) were positive and 87 (41.04%) were negative. Similarly, CD28^−^CD57^+^KLRG1^+^CD8^+^ T cell proportion (T_8_sen) was available for 228 patients with aNSCLC, of which 45 (19.74%) were over 39.5% (T_8_sen^high^) and 183 (80.26%) were lower than 39.5% (T_8_sen^low^).

In the PCT-treated patient cohort, among patients evaluable for response, median progression-free survival (PFS) and overall survival (OS) were 4.98 [95% confidence interval (CI), 4.14 to 7.11] and 8.03 (95% CI, 7.07 to not reached) months, respectively. T_8_sen analysis was performed in 61 patients, of which 11.48% (*n* = 7) were T_8_sen^high^, with a global median of CD8^+^ senescent T cell proportions of 22.54%.

In the PREMIS cohort (ICB-treated), 46.28% of patients received ICB as a first-line treatment. Median PFS and OS were 3.78 (95% CI, 2.76 to 5.75) and 12.89 (95% CI, 9.76 to 19.59) months, respectively. T_8_sen status was available for 120 patients, with a median CD8^+^ senescent T cell proportion of 22.23%. Moreover, 20.66% (*n* = 25) of patients were T_8_sen^high^.

In the CTC cohort (ICB-treated), 87.50% of patients received ICB as a second- or more line of treatment, and 87.50% have been previously treated by chemotherapy. Median PFS and OS were 5.36 (95% CI, 2.01 to 7.99) and 13.18 (95% CI, 7.04 to 24.23) months, respectively. T_8_sen status was available for 47 patients, with a median CD8^+^ senescent T cell proportion of 22.11%. A total of 23.21% (*n* = 13) patients were T_8_sen^high^.

The detailed clinicobiological characteristics of patients in the three cohorts are described in table S1. T_8_sen^high^ patients were older than T_8_sen^low^ patients (median, 71.40 years old versus 62.95 years old; *P* < 0.001); no other difference could be found in the clinical characteristics [body mass index (BMI), sex, and presence of liver metastases].

### Circulating T_8_sen cells are characterized by higher expression of the transcription factor T-bet and senescence-associated β-galactosidase

We have previously shown that T_8_sen cells have a lower proliferative capacity in vitro and that these cells produce high levels of interferon-γ (IFN-γ) and tumor necrosis factor–α (TNF-α) and low levels of IL-2 compared to other CD8^+^ T cell populations (*16*), thus resembling to senescent cells. To determine whether T_8_sen cells display other classical senescence markers, senescence-associated β-galactosidase (SA-βgal) was monitored (*17*). SA-βgal mean fluorescence intensity of CD8^+^ T cell subpopulations was assessed in seven patients. Compared to CD8^+^CD28^+^ T cells, CD8^+^CD28^−^CD57^+^KLRG1^+^ T cells had a higher and significant expression of SA-βgal (fig. S1A).

We also analyzed the expression of key transcription factors T-bet and Eomes. These transcription factors are involved in the regulation of IFN-γ production, and their expressions vary along with the differentiation stage of lymphocytes . We focused our analysis on representative populations of CD8^+^ T cells indicated on the gating strategy (fig. S1B). Eomes expression was higher in CD8^+^KLRG1^+^ and CD8^+^CD28^−^ cells (fig. S1C). T-bet expression was increased in CD8^+^CD28^−^ cells, particularly among the CD57^+^KLRG1^+^ subpopulation (fig. S1D).

Overall, T_8_sen cells have phenotypic (SA-βgal^+^ and T-bet^+^) and functional (IFN-γ^+^, TNF-α^+^, low proliferation, and low IL-2 secretion) (*16*) characteristics of senescent cells which are more pronounced than other CD8^+^ T cell populations. Therefore, these cells are named senescent CD8^+^ T cells hereafter.

### Senescent CD8^+^ T cells are not associated with systemic inflammation

**Fig. 1.**
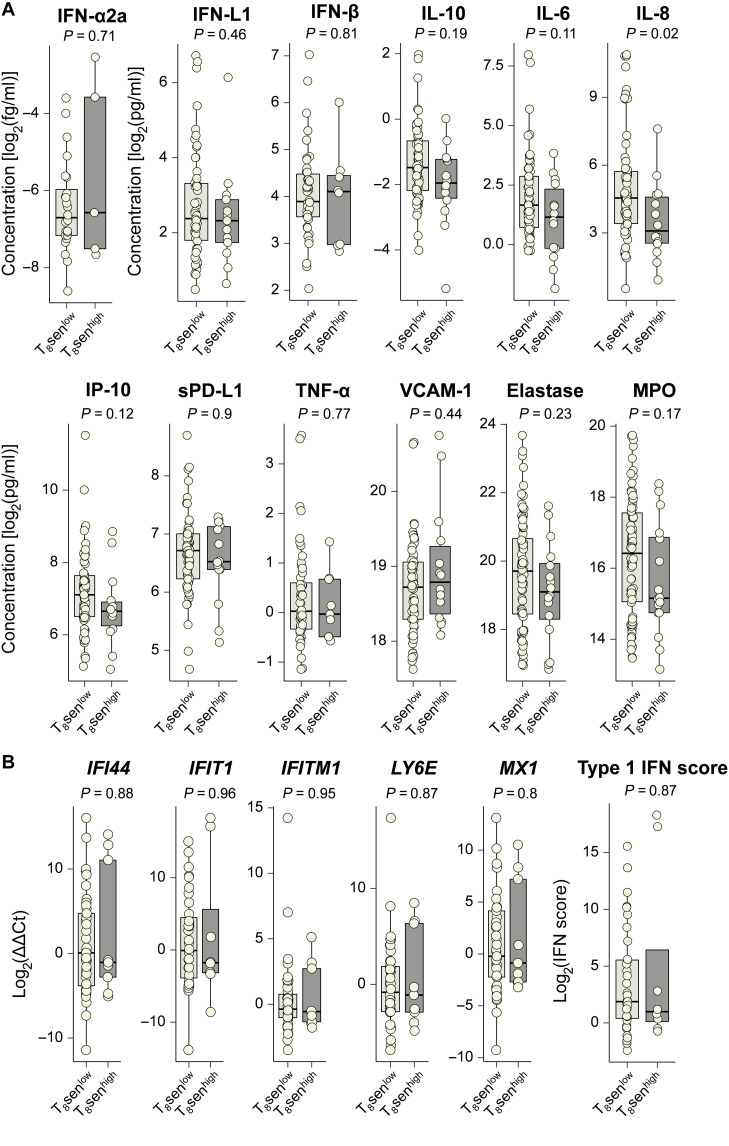
T_8_sen^high^ status is not associated with systemic inflammation, plasmatic oxidative stress, or type I IFN signature. (**A**) Dosage of inflammatory proteins, IFNs, and IFN-associated proteins was assessed on 79 thawed plasma from patients with aNSCLC. Dosage of oxidative stress–associated molecules MPO and elastase was performed on 94 thawed plasma from patients with aNSCLC. Whole-blood immunophenotyping of T_8_sen cells was assessed on the samples. Concentrations of proteins (in pg/ml except for IFN-α in fg/ml) were log_2_-normalized and analyzed according to T_8_sen status. (**B**) Relative expression of ISG and type I IFN score were assessed by RT-qPCR on thawed PBMC from 43 patients with aNSCLC and compared between T_8_sen^high^ and T_8_sen^low^ patients. Differences between groups are analyzed by Mann-Whitney test.

Inflammatory mediators and oxidative stress could be a cause and a consequence of the maintenance of the senescence state. As an example, IFN-γ promotes the maintenance of the p53 pathway, which plays a role in cell cycle arrest, whereas its secretion is also linked to SASP. Type I IFNs are also known to induce lymphocyte dysfunction in chronic viral infections (*18*), with potential decrease of telomerase activity (*19*). We therefore sought to explore the link between blood inflammatory mediators and senescent CD8^+^ T cells in patients with aNSCLC. The plasmatic concentration of 12 soluble factors including several IFNs and associated molecules [IFN-α, IFN-β, IFN-λ, Interferon gamma-induced protein 10 (IP-10), and soluble PD-L1], proinflammatory cytokines and/or described in SASP [IL-6, IL-8, TNF-α, and vascular cell adhesion molecule–1 (VCAM-1)], immunomodulatory cytokine (IL-10), and oxidative stress–associated molecules (elastase and myeloperoxidase) was measured in the plasma of patients with aNSCLC. Among all studied soluble factors, no significant difference between the median plasma concentrations of T_8_sen^low^ and T_8_sen^high^ patients was found except for IL-8 (23.35 versus 8.62; *P* = 0.021; Fig. 1A).

DNA damage response has been shown to promote cellular senescence and secretion of type I IFNs (*20*).We measured the relative expression of five IFN-stimulated genes (ISGs) by reverse transcription quantitative polymerase chain reaction (RT-qPCR) in peripheral blood mononuclear cells (PBMCs) of 43 patients with aNSCLC. Type I IFN score was calculated by the sum of the relative expression of the five ISGs. Overall, no difference was found between relative expressions of *IFI44*, *IFIT1*, *IFITM1*, *LY6E*, and *MX1* between T_8_sen^high^ and T_8_sen^low^ patients, and T_8_sen^high^ patients did not have a higher type I IFN score (median, 2.00 versus 3.66; *P* = 0.87; Fig. 1B).

### CMV is the major viral driver of CD8 T cell senescence in patients with aNSCLC

Chronic viral infections accelerate immune aging, especially T cell compartment, which can acquire senescence-associated markers (*21*, *22*). To explore the association between patient’s viral history and senescent CD8^+^ T cells, a human virome epitope-level antiviral antibody profiling was assessed using the VirScan technology on the serum of 176 patients with aNSCLC.

**Fig. 2.**
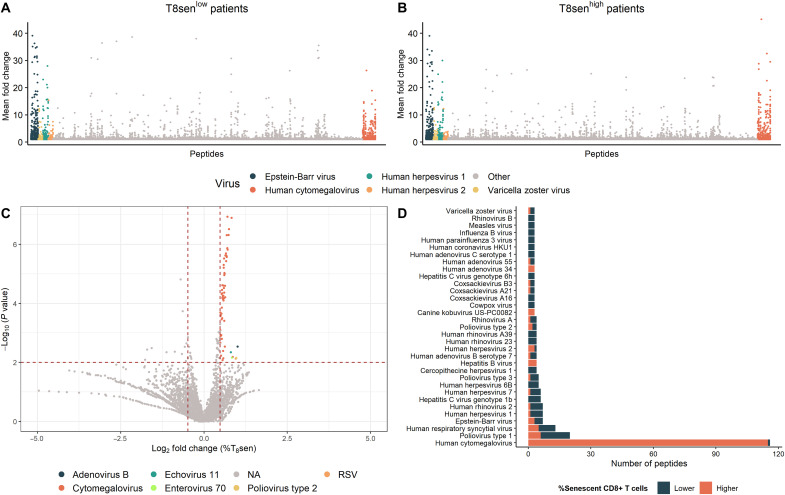
CMV is the main driver of T_8_sen accumulation in patients with aNSCLC. Pan-virus serological profile (VirScan) was assessed in the serum of 176 patients with aNSCLC. (**A** and **B**) Scatter plots representing the mean enrichment of antipeptide antibodies in T_8_sen^low^ (A) and T_8_sen^high^ (B) patients. (**C**) Volcano plot representing the mean percent of T_8_sen in peptide-positive patients divided by that of peptide-negative patient (fold change). Horizontal dotted line defines a *P* value of 0.01 (in the context of multiple testing), and the vertical dotted line represents a meaningful 40% fold change of percentage of T_8_sen cells between peptide-positive and peptide-negative patients (i.e., a log_2_ fold change = ±0.4854). (**D**) Number of viral peptides in which positivity is associated with a higher (orange bars) or lower (blue bars) percentage of CD8^+^ senescent T cells (Wilcoxon *P* < 0.05).

The overall antibody response to viral peptides, including the response against very common viruses in humans like those of the Herpesviridae family, was similar in T_8_sen^high^ and T_8_sen^low^ patients, except for CMV for which the average reactivity seemed higher in T_8_sen^high^ patients (Fig. 2, A and B). For each peptide, the mean proportion of CD8^+^ senescent T cells among peptide-seropositive patients was divided by that of peptide-seronegative patients. Antibodies directed against 74 peptides were significantly associated with a fold change of 40% or more. Of these antiviral antibodies, the majority recognized CMV peptides (55 of 60; 91.6%; Fig. 2C). Then, for each virus, the number of peptide in which seropositivity was associated with a significant enrichment in the CD8^+^ senescent T cell percentages was calculated. Among 33 viruses with at least three significant peptide seroreactivities (*P* ≤ 0.05), CMV dominates with 115 antipeptide antibodies associated with higher proportions of CD8^+^ senescent T cells (Fig. 2D).

**Fig. 3.**
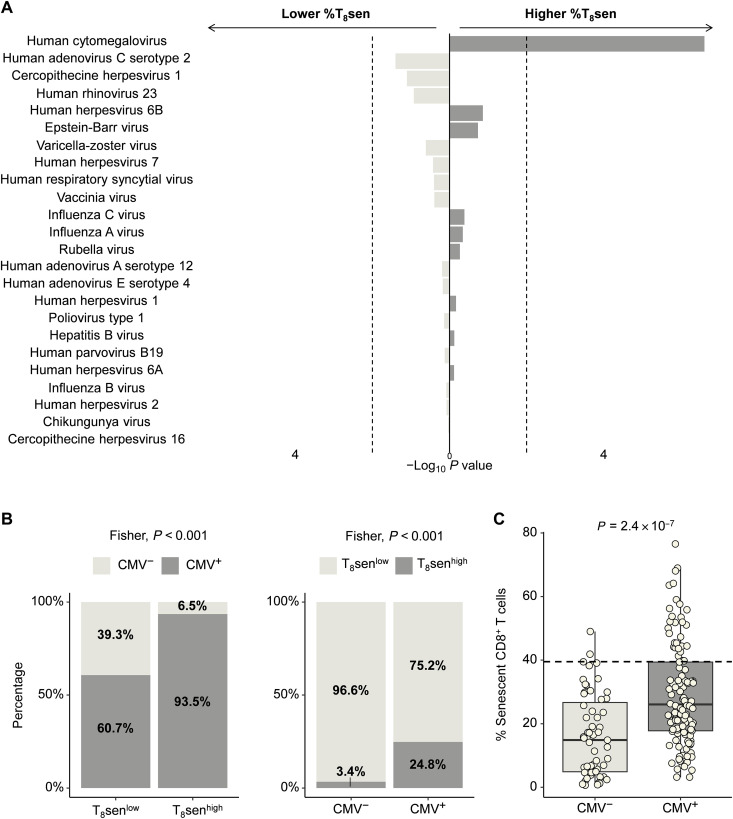
T_8_sen^high^ status is associated with CMV^+^ status. Pan-virus serological profile (VirScan) was assessed in sera of 176 patients with aNSCLC. Virus reactivity was determined for at least reactivity against four viral proteins. Using this threshold, the seropositivity of patients was determined for all virus of the VirScan assay. (**A**) Negative (left) or positive (right) variation of T_8_sen according to viral seropositivity. Only viruses with a minimum of five seropositive patients are represented. The vertical dotted line represents a *P* value of 0.05. (**B**) Proportions of CMV^+^ patients are represented according to T_8_sen status. Proportions of T_8_sen^high^ patients are represented according to CMV status. (**C**) Rate of CD8^+^ senescent T cells among CD8^+^ cells is compared between CMV^+^ and CMV^−^ patients. The horizontal dotted line represent a proportion of CD8^+^ senescent T cells of 39.5% among CD8^+^ cells. Categorical variables (T_8_sen^high/low^, CMV^+/−^) were compared by Fisher test; continuous variables were compared in two populations by Mann-Whitney *t* test, and two continuous variables were compared using Spearman correlation.

Clinical laboratory data of CMV serology were determined in 151 patients with aNSCLC with VirScan data. CMV serology was positive in 61.59% (93 of 151) of patients. We determined a threshold of at least four recognized CMV proteins with the VirScan technology to consider a seropositivity (area under the curve = 0.957, sensitivity = 97.85%, and specificity = 87.93%; fig. S2). Using this threshold, the number of seropositive patients for each virus is detailed in table S2. We then analyzed the proportions of CD8^+^ senescent T cells between virus-positive and virus-negative patients and found that CMV was the only virus associated with a higher proportion of CD8^+^ senescent T cells (Fig. 3A). Majority of T_8_sen^high^ patients (*n* = 29 of 31; 93.5%) were CMV^+^, compared to T_8_sen^low^ (*n* = 88 of 145; 60.7%; *P* < 0.001), while only 24.8% (*n* = 29 of 117) of CMV^+^ patients were T_8_sen^high^ (Fig. 3B). The proportions of T_8_sen cells were significantly higher in CMV^+^ patients (*P* = 2.4 × 10^−7^; Fig. 3C). These data strongly suggest that CMV^+^ status is necessary but not sufficient to switch toward a T_8_sen^high^ status. Regarding other demographic covariates known to affect CMV infection (*23*), a correlation was observed between the country of birth and CMV seropositivity: Patients born outside France were almost all CMV^+^ [*n* = 22 of 23 (95.65%) versus *n* = 103 of 187 (55.08%); *P* < 0.001]. However, considering only CMV^+^ patients, we found no difference in the median %T_8_sen depending on the country of birth (median, 32.82% versus 26.62% for France and others; *P* = 0.42). No difference in the CMV seroprevalence or in the %T_8_sen could be observed regarding the residency department (*P* = 0.75).

To determine whether the impact of CMV on lymphocyte senescence was specific to lung cancer, we measured the T_8_sen status in thawed PBMCs from 51 patients with advanced renal cell carcinoma (RCC) of the PREMIS cohort with CMV serology. In CMV^+^ patients with RCC, the proportions of T_8_sen^high^ patients and T_8_sen cells were significantly higher in CMV^+^ patients (*P* = 0.005 and *P* = 0.00048; fig. S3, A and B), and only two T_8_sen^high^ patients were CMV^−^. These results indicated that the impact of CMV on the accumulation of senescent CD8^+^ T lymphocytes is not restricted to patients with NSCLC.

We then sought to determine whether coinfections with other virus could be associated with accumulation of senescent CD8^+^ T cells in CMV^+^ patients. No other viral coinfection was associated with a greater accumulation of senescent T cells in CMV^+^ patients (fig. S4A). The majority of CMV^+^ patients were seropositive for Epstein-Barr virus (EBV; 116 of 117); it is therefore difficult to conclude on the role of EBV coinfection in the accumulation of senescent T lymphocytes. However, in EBV^+^CMV^−^ patients, barely no patients were T_8_sen^high^, demonstrating that EBV is not a driver factor for the accumulation of T_8_sen (fig. S4B). Together, these data suggest that CMV is a major viral driver of CD8^+^ T cell senescence in patients with aNSCLC.

### T_8_sen^high^ status is not associated with a polyclonal antibody response against CMV nor with concomitant CMV viral load

**Fig. 4.**
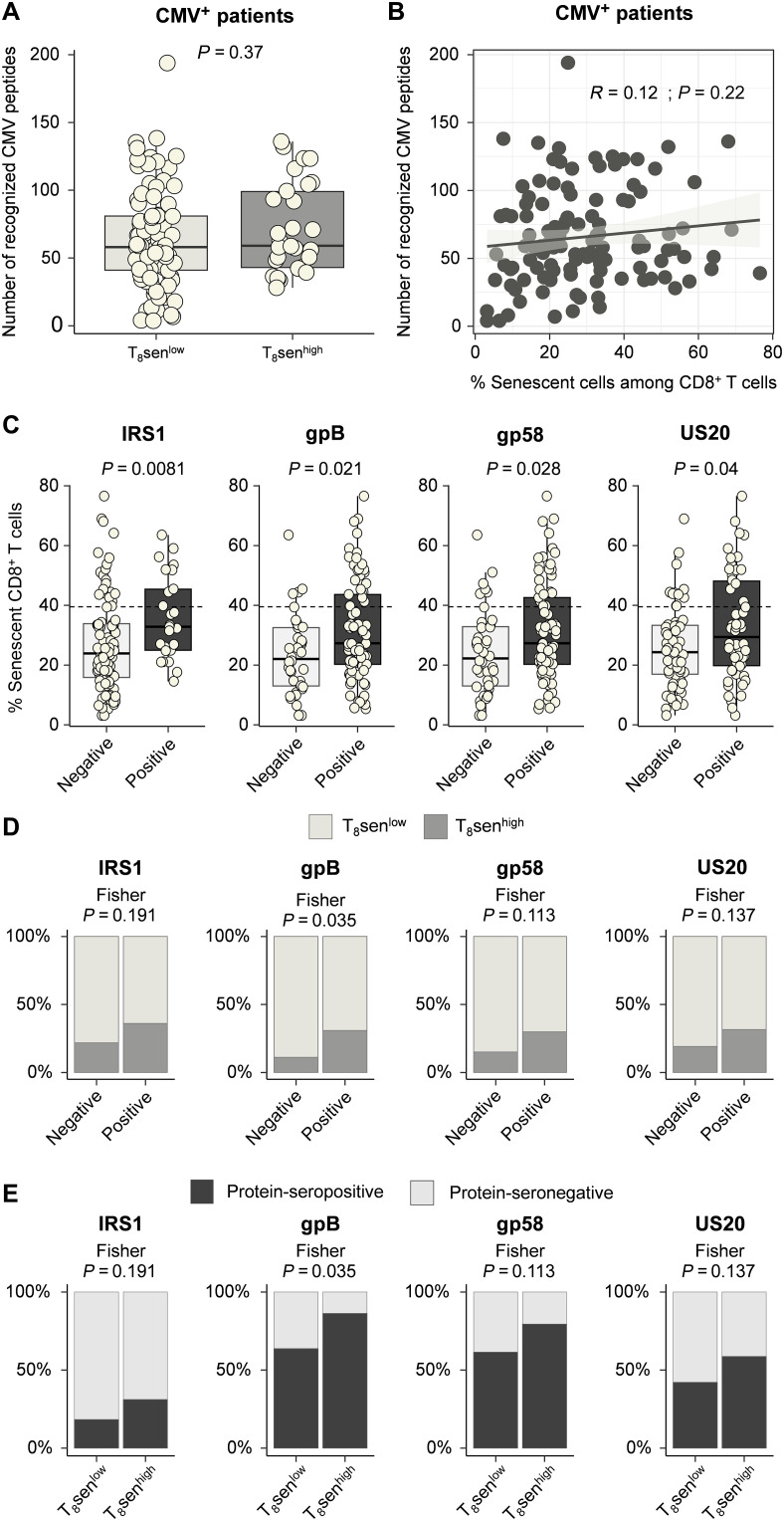
T_8_sen^high^ status is not associated with a polyclonal profile of immunization against CMV. Pan-virus serological profile (VirScan) was assessed in sera of 176 patients with aNSCLC. (**A** and **B**) Among 117 CMV^+^ patients, the number of recognized CMV peptides is represented between T_8_sen^high^ and T_8_sen^low^ patients (A) and according to the proportion of CD8^+^ senescent T cells among CD8^+^ (B). (**C**) Proportion of CD8^+^ senescent T cells depending on the presence of antibodies targeting IRS1, gB, gp58, and US20 among CMV^+^ patients in the VirScan cohort. (**D**) Proportions of T_8_sen^high^ and T_8_sen^low^ patients depending on each protein’s seropositivity in CMV^+^ patients. (**E**) Proportions of IRS1-, gB-, gp58-, and US20-positive patients according to T_8_sen status in CMV^+^ patients. Categorical variables were compared by Fisher test; continuous variables were compared in two populations by Mann-Whitney *t* test, and two continuous variables were compared using Spearman correlation.

To identify other factors favoring the switch to a strong accumulation of senescent T lymphocytes, i.e., greater than 39.5% according to our cut-off value (T_8_sen^high^), we investigated the CMV immunization profile. Among 117 CMV^+^ patients, the diversity of antibodies targeting different CMV epitopes was not greater in T_8_sen^high^ patients (*P* = 0.37; Fig. 4A) and did not correlate with the proportion of senescent CD8^+^ T cells (*R* = 0.12, *P* = 0.22; Fig. 4B). We then analyzed the serological profile toward CMV proteins most frequently recognized by patients (at least 20% protein-seropositive patients and 20% protein-seronegative patients; *n* = 29 proteins). Median CD8^+^ senescent T cell proportions were analyzed between protein-seropositive and protein-seronegative patients. Among these proteins, although reactivity to tegument protein from internal repeat sequence 1 (IRS1), glycoprotein B (gB), glycoprotein 58 (gp58), and membrane protein from unique short region 20 (US20) proteins was associated with a significantly higher proportion of CD8^+^ senescent T cells (Fig. 4C), only reactivity against gB was significantly associated with higher proportions of T_8_sen^high^ patients (Fig. 4D). Likewise, T_8_sen^high^ patients were significantly more reactive against gB (Fig. 4E and Table 1). These data suggest that T_8_sen^high^ status is not determined by a polyclonal antibody response directed against several CMV epitopes but rather by a more restricted response to certain epitopes.

**Table 1.** T_8_sen^high^ status is not associated with specific CMV-protein immunization. Pan-virus serological profile was assessed in sera of 176 patients with aNSCLC. Protein reactivity was positive if at least one peptide was recognized (enrichment fold change > 1). In CMV^+^ patients (*n* = 117), proportions of CD8^+^ senescent T cells were analyzed according to reactivity against 29 proteins with at least 20% of protein-seropositive patients and 20% of protein-seronegative patients, by Mann-Whitney test. Proportions of protein-seropositive patients were then compared between T_8_sen^high^ and T_8_sen^low^ patients by Fisher’s test. T_8_sen, senescent CD8^+^ T cells defined by the proportion of CD28^−^CD57^+^KLRG1^+^ T cells among CD8^+^; US, unique short; UL, unique long. Bolded values, *P* < 0.05.

Protein	Number and [proportion] of protein-seropositive patients	Median CD8^+^ senescent T cell proportions			Proportions of protein-seropositive patients		
		Seronegative patients	Seropositive patients	*P* value	In T_8_sen^low^ patients	In T_8_sen^high^ patients	*P* value
Tegument protein IRS1	25 [21.37%]	23.895	32.81	**0.0081**	18.18	31.03	0.1906
gB (fragment)	81 [69.23%]	22.035	27.27	**0.0208**	63.64	86.21	**0.0352**
Glycoprotein gp58 (fragment)	77 [65.81%]	22.215	27.27	**0.0275**	61.36	79.31	0.1132
Membrane protein US20	54 [46.15%]	24.31	29.35	**0.0401**	42.05	58.62	0.1371
Transmembrane protein HWLF3	39 [33.33%]	24.885	31.52	0.0528	30.68	41.38	0.3642
Capsid protein P40	40 [34.19%]	25.68	28.435	0.1208	31.82	41.38	0.3727
gB amino part of (fragment)	33 [28.21%]	25.225	30.88	0.1284	26.14	34.48	0.4759
Tegument protein UL83 (pp65)	56 [47.86%]	24.87	31.49	0.1429	44.32	58.62	0.2038
UL139	72 [61.54%]	21.42	26.99	0.1681	59.09	68.97	0.3858
Matrix phosphoprotein (fragment)	56 [47.86%]	24.81	28.435	0.2311	45.45	55.17	0.3975
IE19	50 [42.74%]	25.58	27.41	0.2977	43.18	41.38	1.0000
Protein IRL10	38 [32.48%]	25.72	26.62	0.2988	29.55	41.38	0.2591
UL36	32 [27.35%]	26.06	26.38	0.4374	25.00	34.48	0.3431
UL132 (fragment)	58 [49.57%]	26.26	25.89	0.5597	46.59	58.62	0.2904
Protein ICP36	45 [38.46%]	25.65	27.74	0.5658	38.64	37.93	1.0000
G-protein coupled receptor homolog US27	27 [23.08%]	26.99	25.68	0.6070	22.73	24.14	1.0000
Immediate-early protein 1 (IE1)	59 [50.43%]	25.99	26.06	0.6826	51.14	48.28	0.8328
Envelope glycoprotein UL132	69 [58.97%]	25.265	27.08	0.7229	56.82	65.52	0.5150
Tegument protein UL11 homolog (pp28)	92 [78.63%]	27.08	25.7	0.7521	78.41	79.31	1.0000
Viral transcription factor IE2 (UL122)	75 [64.1%]	27.225	24.87	0.7611	63.64	65.52	1.0000
UL132B	63 [53.85%]	26.58	25.68	0.7678	51.14	62.07	0.3913
UL25 (pp85)	33 [28.21%]	25.99	26.06	0.7852	27.27	31.03	0.8123
Tegument protein UL82 (pp71)	33 [28.21%]	26.58	25.58	0.8180	29.55	24.14	0.6413
IE9	47 [40.17%]	26.58	25.68	0.8218	40.91	37.93	0.8300
Immediate-early protein (fragment)	44 [37.61%]	26.26	25.87	0.8373	37.50	37.93	1.0000
Immediate-early protein 2 (IE2)	80 [68.38%]	27.08	25.32	0.8489	67.05	72.41	0.6515
Early phosphoprotein p84	25 [21.37%]	26.16	25.68	0.9021	21.59	20.69	1.0000
Membrane protein RL12	40 [34.19%]	25.72	26.48	0.9199	35.23	31.03	0.8222
Tegument protein UL71	67 [57.26%]	27.6	24.96	0.9232	56.82	58.62	1.0000

To clarify the relationship between EBV infection and T_8_sen status, we analyzed the EBV serological profile. No reactivity against any EBV protein was associated with an increase in T_8_sen; Epstein-Barr virus nuclear antigen 1 (EBNA 1) and gM-seropositive patients had even lower levels of T_8_sen. In T_8_sen^high^ patients, we did not observe a different reactivity profile (table S3) contrary to CMV reactivity profile, again suggesting that EBV is not a driver of T cell senescence in our study.

Last, concomitant CMV viral load was assessed using qPCR in the plasma of 37 patients. CMV DNA could not be detected in any sample of T_8_sen^high^ or T_8_sen^low^ patients, which does not argue for a poorer control of CMV replication in T_8_sen^high^ patients at least at the time of measurement.

### CMV-specific T cells accumulate in Tsen compartment

**Fig. 5.**
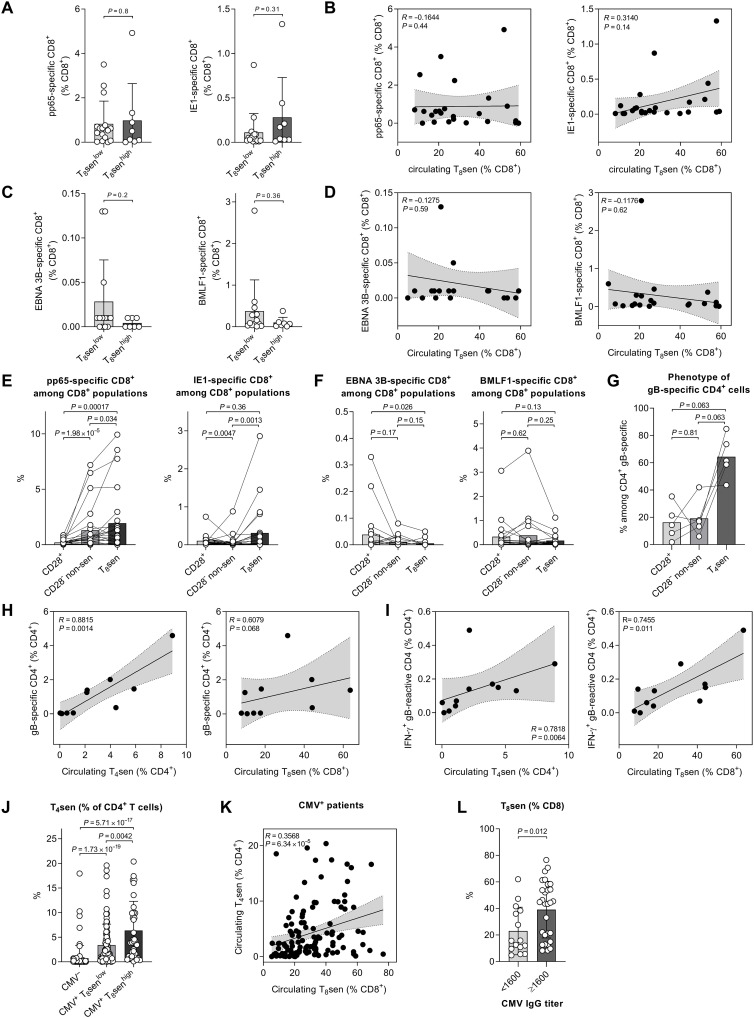
CMV-specific T cells accumulated in the Tsen compartment. Identification of CMV (pp65 and IE1 epitopes)– or EBV (EBNA 3B and BMLF1 epitopes)–specific CD8^+^ T cells was assessed in 24 CMV^+^ and 20 EBV^+^ patients with aNSCLC. Identification of gB-specific CD4^+^ T cells and stimulation of PBMC with gB peptide for IFN-γ staining were performed in 10 and 11 patients with aNSCLC. Anti-CMV IgG titer was determined in sera or plasma from 44 patients with aNSCLC. Proportions of pp65- and IE1-specific CD8^+^ cells depending on (**A**) T_8_sen status and (**B**) proportions of circulating T_8_sen. Proportions of EBNA 3B– and BMLF1-specific CD8^+^ cells depending on (**C**) T_8_sen status and (**D**) proportions of circulating T_8_sen. Proportions of (**E**) pp65- and IE1-specific CD8^+^ cells and (**F**) EBNA 3B– or BMLF1-specific CD8^+^ cells, among CD8^+^ populations: CD28^+^, CD28^−^ non-sen (CD57^−^KLRG1^−^, CD57^+^KLRG1^−^, and CD57^−^KLRG1^+^), and T_8_sen. (**G**) Proportions of CD28^+^, CD28^−^ non-sen, and T_4_sen cells among gB-specific CD4^+^ cells. Proportions of (**H**) gB-specific CD4^+^ cells and (**I**) IFN-γ^+^ gB-reactive CD4^+^ cells, according to proportions of circulating T_4_sen and T_8_sen. (**J**) Proportions of T_4_sen depending on CMV and T_8_sen status. (**K**) In CMV^+^ patients, correlation between T_4_sen and T_8_sen. (**L**) Proportions of T_8_sen depending on CMV IgG titer. Continuous variables were compared in two populations by Mann-Whitney (unpaired) and Wilcoxon (paired) *t* test. Two continuous variables were compared using Spearman correlation T_4_sen: CD4^+^CD28^−^CD57^+^KLRG1^+^.

To determine whether the enrichment of senescent CD8^+^ T cells was associated with the inflation of CMV- or EBV-specific CD8^+^ pools, we measured the proportions of CD8^+^ cells specific for two immunodominant CMV peptides [phosphoprotein 65 (pp65) and immediate early protein 1 (IE1)] and two EBV peptides [BamHI-M leftward reading frame 1 (BMLF1) and Epstein-Barr virus nuclear antigen 3B (EBNA 3B)] in HLA-A*0201 CMV^+^ (*n* = 24) or EBV^+^ (*n* = 20) patients with aNSCLC (fig. S5A). The levels of pp65- and IE1-specific CD8^+^ T cells were not significantly higher in T_8_sen^high^ patients compared to T_8_sen^low^ patients (Fig. 5A), and they were not correlated with circulating T_8_sen levels (Fig. 5B). Among 20 EBV^+^ patients, the levels of EBNA 3B– and BMLF1-specific T CD8^+^ cells were not higher in T_8_sen^high^ patients compared to T_8_sen^low^ patients (Fig. 5C), and these levels were not correlated with circulating T_8_sen levels (Fig. 5D). However, IE1- and pp65-specific CD8^+^ T cells accumulated preferentially within the T_8_sen compartment (Fig. 5E). In marked contrast, EBV-specific CD8^+^ T cells did not accumulate in the T_8_sen compartment but rather in CD8^+^ CD28^+^ T cells (Fig. 5F).We then focused on CMV-specific CD4^+^ T cells by performing a gB tetramer staining in 10 samples from HLA-DRB1*0701 patients (fig. S5B). gB-specific CD4^+^ T cells were predominantly CD28^−^CD57^+^KLRG1^+^ (T_4_sen; Fig. 5G) and were highly correlated with circulating T_4_sen levels (*r* = 0.8815); in other words, most T_4_sen were specific for gB. A correlation trend was also observed between gB-specific CD4^+^ T cells and T_8_sen levels (*r* = 0.6079; Fig. 5H). Proportions of IFN-γ^+^ gB-reactive CD4^+^ T cells were correlated with circulating T_4_sen (*r* = 0.7818; Fig. 5I, left) and T_8_sen levels (*r* = 0.7455; Fig. 5I, right). These data suggest an accumulation of CMV-specific T cells within the senescent compartment, with a link between the accumulation of gB-specific senescent CD4^+^ cells and T_8_sen cells. In patients with aNSCLC, T_4_sen levels were higher in CMV^+^ patients, particularly in T_8_sen^high^ (Fig. 5J) and were correlated with T_8_sen levels in CMV^+^ patients (Fig. 5K).

Because accumulation of gB-specific CD4^+^ T cells and gB-specific antibodies were associated with enrichment of T_8_sen, we analyzed the anti-CMV immunoglobulin G (IgG) titers. In patients with an anti-CMV IgG titer of at least 1/1600, the proportions of T_8_sen were significantly higher (Fig. 5L).

Together, these results demonstrated that enrichment of T_8_sen cells is observed in patients whose T cells recognize a MHC II–restricted epitope from CMV. These data provide a more direct mechanistic tie between CMV infection and enrichment of T_8_sen.

### Higher CD8 T cell senescence in advanced tumor is dependent on CMV seropositivity

**Fig. 6.**
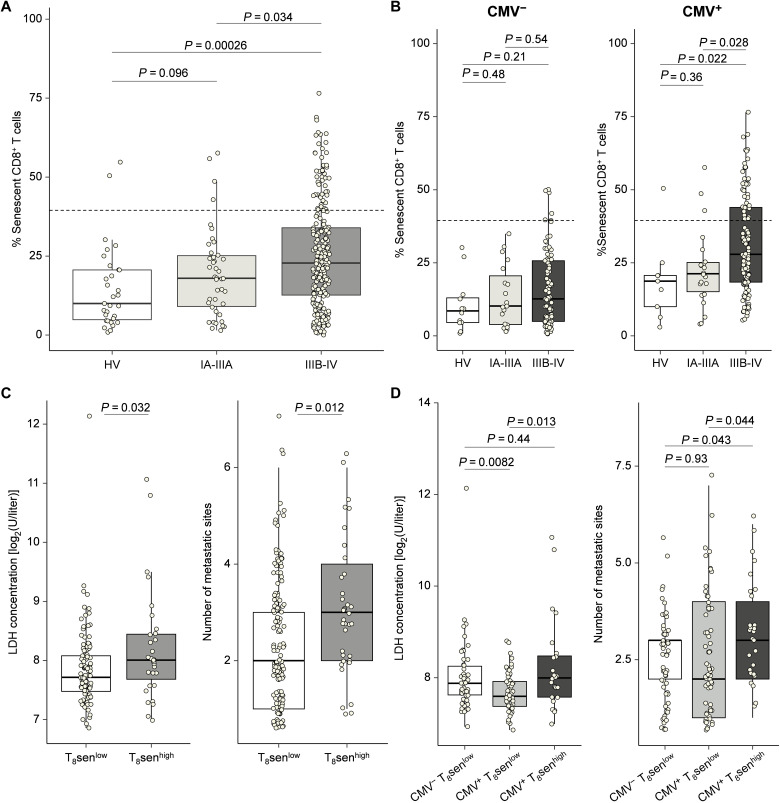
Higher proportions of CD8^+^ senescent T cells in NSCLC advanced stages only in CMV^+^ patients. T_8_sen status was assessed by flow cytometry in 282 patients with NSCLC with tumor stage available and 31 healthy volunteers (HV; *n* = 21 with CMV status). (**A**) Proportions of CD8^+^ senescent T cells depending on NSCLC stage. (**B**) Proportions of CD8^+^ senescent T cells depending on NSCLC stage and CMV status. (**C**) LDH concentrations and number of metastatic sites according to T_8_sen status. (**D**) LDH concentrations and number of metastatic sites according to CMV seropositivity and T_8_sen status. Continuous variables were compared between populations by Mann-Whitney *t* test.

To assess the relationship between the proportion of senescent CD8^+^ T cells and the presence of a malignancy, immune phenotyping of senescent CD8^+^ T cells was performed by flow cytometry on whole-blood samples from 282 patients with NSCLC for which tumor stage was available (*n* = 42 stage IA-IIIA NSCLC; *n* = 240 stage IIIB-IV NSCLC) and 31 healthy volunteers. As shown in Fig. 6, senescent CD8^+^ T cells were significantly higher in advanced (IIIB/IV) stages compared to early (IA/IIIA) stages only in CMV^+^ patients (Fig. 6, A and B). Among the patients with a locally advanced or metastatic stage (IIIB/IV), patients with a T_8_sen^high^ status had higher lactate dehydrogenase (LDH) levels and a greater number of metastatic sites. Thus, the T_8_sen^high^ status seems to be associated with tumor burden (Fig. 6C). After adjusting for CMV status, only CMV^+^ T_8_sen^high^ patients have a significant increase in LDH and in the number of metastatic sites (Fig. 6D). Overall, even in patients with aNSCLC, the CMV status is necessary to observe higher proportions of T_8_sen, this enrichment being greater in patients with high tumor burden.

### CMV-induced T cell senescence is associated with poorer outcomes in patients with aNSCLC treated by ICB

**Fig. 7.**
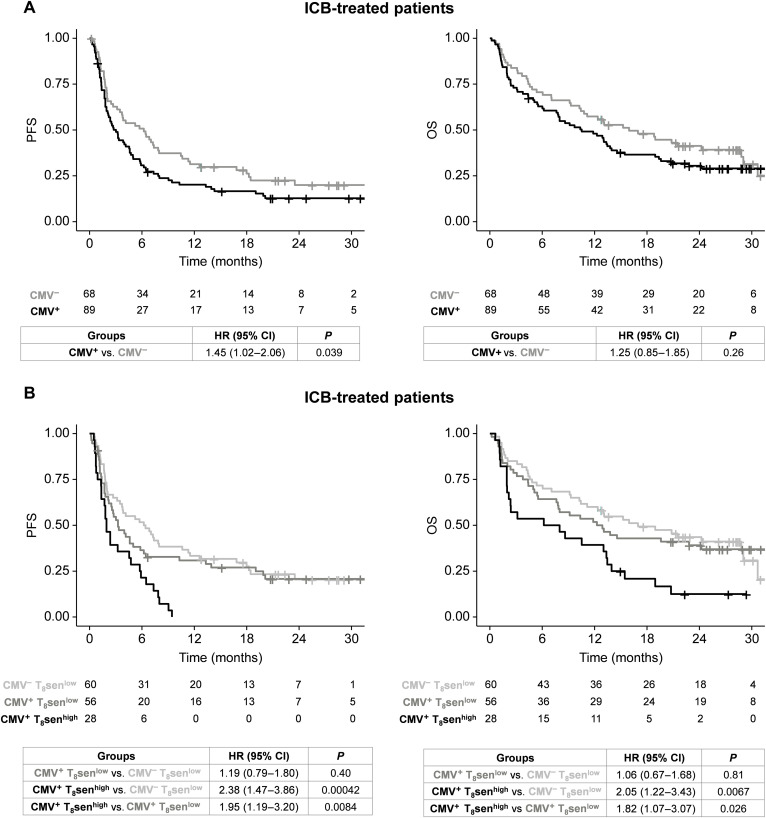
CMV-induced senescent immune phenotype is associated with poorer outcome in patients with aNSCLC treated by ICB. In 157 patients with aNSCLC treated by ICB, (**A**) probability of PFS and OS depending on CMV status and (**B**) PFS and OS depending on CMV and T_8_sen status. Survival curves were analyzed using a Cox regression model.

Considering the fact that CMV is associated with T_8_sen status, which predicts resistance to ICB (*16*), we evaluated CMV status impact on outcomes in patients with aNSCLC treated with ICB. When pooling all ICB-treated patients with advanced NSCLC (*n* = 157), CMV seropositivity was associated with poorer PFS (median PFS, 3.02 months versus 6.21 months in CMV^−^, *P* = 0.039) but had no clear impact on OS (median OS, 10.52 months versus 15.95 months in CMV^−^, *P* = 0.26; Fig. 7A). Furthermore, CMV status did not predict PFS nor OS in PCT-treated patients (fig. S6A). However, after adjustment on T_8_sen status in ICB-pooled cohorts, CMV serology was not associated with PFS [Hazard Ratio (HR) for CMV: 1.19 (95% CI, 0.79 to 1.80; *P* = 0.40); HR for T_8_sen status was 2.00 (95%CI, 1.23 to 3.25; *P* = 0.0054)], indicating that the effect of CMV on survival likely only relies on the underlying T_8_sen status. Furthermore, no difference in PFS or OS was observed between CMV^+^ T_8_sen^low^ and CMV^−^ T_8_sen^low^ patients [HR = 1.19 (95% CI, 0.79 to 1.80; *P* = 0.40) and HR = 1.06 (95%CI, 0.67 to 1.68; *P* = 0.81), respectively; Fig. 7B]. Last, the deleterious effect of T_8_sen^high^ status is specific to ICB treatment, as it does not predict outcomes in PCT-treated patients, for which a trend toward a better OS was even seen in CMV^+^ patients (fig. S6B). As CMV was associated with the country of birth (France versus elsewhere) in our cohort, we investigated whether T_8_sen could be a surrogate variable for socioeconomic conditions. After adjustment on the country of birth, T_8_sen^high^ status remained associated with poorer PFS in ICB-treated patients (HR: 1.9, *P* = 0.0018) or OS (HR = 1.91, *P* = 0.0035), whereas the place of birth did not seem to have an impact (HR = 1.04, *P* = 0.87 for PFS; HR = 1.08, *P* = 0.78 for OS). All in all, these data indicate that the impact of CMV status in ICB-treated patients is driven by the enrichment in CD8^+^ senescent T cells.

## DISCUSSION

Chronic viral infections are known to accelerate immune aging and to induce immune senescence (*21*, *22*). We have shown that among 206 viruses with human tropism, only CMV seropositivity was associated with senescence of CD8^+^ T cells in patients with aNSCLC. Excluding CMV, the antiviral serological profile appears similar between T_8_sen^low^ and T_8_sen^high^ patients, including the response against the Herpesviridae family. To our knowledge, no study has demonstrated the major viral role of CMV in the accumulation senescence of T cells in patients with aNSCLC. While the majority of T_8_sen^high^ patients were CMV seropositive, only 24.8% of CMV^+^ patients were T_8_sen^high^, meaning that CMV^+^ status is a necessary viral driver but is not sufficient to acquire T_8_sen^high^ status.

Focusing on the anti-CMV serological profile in CMV^+^ patients, we did not observe a higher diversity of targeted CMV epitopes in T_8_sen^high^ patients, suggesting that CD8^+^ T cell senescence is not associated with a polyclonal response against CMV. In CMV^+^ patients, the reactivity toward IRS1, gB, gp58, and US20 was associated with a significantly higher proportion of CD8^+^ senescent T cells.

It has been suggested that the genetic variability of human CMV (HCMV) can influence immune escape and have an impact on viral pathogenesis (*24*). The US20 protein is known to induce escape from the natural killer (NK) response by down-regulating NKG2DL and MICA (*25*) and to promote virus replication in endothelial cells (*26*). The viral protein IRS1 promotes the survival of the infected cell by inhibiting autophagy (*27*). In CMV^+^T_8_sen^high^ patients, the persistence of an HCMV strain overexpressing US20 and IRS1 could reduce the ability of NK cells to target CMV-infected cells then favoring a chronic and active T cell response against these cells, leading to T cell senescence. gB is a leading vaccine candidate and the most advanced in terms of vaccine development; gB is one of the most abundant proteins within the viral envelope and important for viral entry (*28*, *29*). A marked dominance of CD4^+^ T cell responses against gB is observed in CMV^+^ donors in comparison with much weaker induction of gB-CD8^+^ T cell responses. CD4^+^ T cells recognizing gB-derived epitopes displayed a much greater cytotoxic potential with very strong production of IFN-γ, TNF-α, and granzyme B and low capacity to produce IL-2 compared to other CMV-specific CD4^+^ T cells (*30*), thus having functional similarities with T_8_sen cells. According to previous data, we showed that the majority of gB-specific CD4^+^ T cells were CD28^−^CD57^+^KLRG1^+^ (T_4_sen). Moreover, gB-specific and gB-reactive CD4^+^ T cells correlated with T_8_sen. Thus, immunodominant gB-specific CD4^+^ T cell response could be the key factor participating in a shift toward more global lymphocyte senescence.

As CMV seems to be the unique viral driver of senescence, coinfection with another virus could be the second hit needed to acquire a T_8_sen^high^ status. Infection by latent viruses such as HIV and EBV may lead to the expansion of viral epitope–specific T cells expressing senescence markers (*21*). Similarly, it has been shown that T cells from HIV^+^ patients strongly express the senescence marker p16 in an age-independent manner in untreated patients (*31*). Resolved infections without latent stage can also induce senescence, especially in the elderly, such as influenza virus–specific T cells expressing CD57, KLRG1, and T-bet markers (*32*). However, an effect of coinfections on the levels of senescent T cells could not be observed in CMV^+^ patients in our cohort. These results suggested that viral coinfections of CMV^+^ patients could not explain T_8_sen^high^ status in patients with NSCLC.

A limitation of our study should be pointed out. Concerning the VirScan assay, although the threshold that we determined for virus positivity (a minimum of four positive proteins) is close to what has been described elsewhere (*33*), it might lead to an underdetection of viruses with shorter genome. Moreover, despite a large number of patients included (*n* = 176), our study may not have the power to detect association between CD8 T cell senescence and viruses that less frequently infect humans or viruses that are present in nearly all patients (5 of 176 screened patients only were negative to EBV). Another limitation could be that antibody binding to linear epitopes and specificities toward epitopes in natural tertiary structures may have been underdetected. Nevertheless, results obtained with VirScan assay are consistent with CMV serology both in the literature (*33*) and in our study, highlighting the robustness of the method.

Aging is associated with a loss of control of viral replication in old patients and more viral reactivation (*34*) that could promote T cell senescence by chronic antigenic stimulation. In our work, no patient had detectable CMV DNA, which does not pledge for a loss of control of viral replication in T_8_sen^high^ patients. Our data only relate to viral replication at a particular moment and does not reflect the number of reactivations that each patient may have experienced. To better address this question, we measured anti-CMV IgG titers because high titers were associated with a higher risk of developing CMV reactivation (*35*, *36*). We demonstrated that higher titers were associated with enrichment of T_8_sen, suggesting repetitive immunological stimulation by CMV in T_8_sen^high^ patients. CMV reactivations can also be triggered by several factors, such as chronic proinflammatory state or oxidative stress. Inflammaging plays a major role in the development of metabolic age-related diseases such as type 2 diabetes, where insulin resistance or obesity are associated with chronic proinflammatory state, oxidative stress, and accumulation of senescent T cells (*37*, *38*). Obese patients may, in turn, experience more HCMV reactivations, but we did not observe an association between BMI and T_8_sen status in patients with aNSCLC. The metabolic profile of patients should be considered in future experiments.

We have also demonstrated that the expression of transcription factor T-bet was higher in T_8_sen cells compared to nonsenescent CD8^+^ T cells. It has been shown that T-bet was induced during the primary response against CMV with sustained expression in HCMV-specific T cells during latency with up-regulation of IFN-γ–regulated genes (*39*). Middle-aged and elderly CMV-seropositive patients have increased levels of CD8^+^ T-bet^+^ T cells in comparison to young CMV-seropositive donors (*40*). Together, these results suggest shared phenotypic and functional characteristics between T_8_sen population and CMV-specific T cells.

Systemic inflammation is known to induce both CMV reactivation (*41*) and immunosenescence, with a major role of the SASP in promoting and maintaining immune aging (*8*). Unexpectedly, plasmatic IL-8 was decreased in T_8_sen^high^ patients. In addition to its proinflammatory role, IL-8 was shown to support the senescence cell cycle arrest through autocrine and paracrine mechanisms (*42*). Thus, lower IL-8 concentrations in T_8_sen^high^ patients could reflect an active consumption. We did not observe an increase in any other plasmatic inflammatory protein (IL-6, IP-10, and TNF-α) in T_8_sen^high^ patients either, raising the possibility of “nonclassical” systemic factors, which could be associated to senescence. Prior studies have shown a negative correlation between plasmatic levels of Receptor activator of nuclear factor kappa-Β ligand (RANKL) and the proportions of CD8^+^ effector memory re-expressing CD45RA (TEMRA) CD28^−^ (*43*). Similarly, one study identified CXCL9 as the main contributor to patients’ “inflammatory” clock-aging (iAge), which, in turn, was inversely correlated to naïve CD8^+^ T cells (*44*). Oxidative stress is also known not only to induce DNA damage and telomere shortening but also to promote CMV reactivations (*45*). No difference was observed in the plasmatic levels of myeloperoxidase and elastase between T_8_sen^high^ and T_8_sen^low^ patients, raising the potential involvement of more conventional markers of oxidative stress in the induction of stress-related senescence.

Latent viral infections were shown to be associated with type I IFN signature, which is essential for the control of viral replication in the acute phase but deleterious when chronicizing by promoting lymphocyte dysfunction (*18*, *46*). This balance between protective and deleterious role of IFN is called the “IFN paradox,” which has largely been described in HIV-seropositive patients. Several studies have demonstrated the capacity of IFN-α to inhibit telomerase activity through a down-regulation of the catalytic subunit human telomerase reverse transcriptase (hTERT) or an increase in p38/mitogen-activated protein kinase signaling, thus promoting cellular senescence (*19*). We assessed the relative expression of five ISG for calculation of type I IFN score and quantified plasmatic IFN-associated proteins. None of these genes and proteins were increased in aNSCLC T_8_sen^high^ patients, and T_8_sen^high^ patients did not have a higher type I IFN score. One might ask whether this IFN paradox is really applicable in the case of CMV infection, especially given that the HCMV genome contains many genes encoding proteins involved in silencing IFN signaling. For example, *UL111A*, which encodes vIL-10, inhibits IFN-α and β transcription in plasmacytoid dendritic cell (*47*), and *UL35* and *UL83* encode pUL35 and pp65, known to down-regulate type I IFN signaling by inactivating the DNA sensor cyclic GMP‐AMP synthase (cGAS) (*48*, *49*). The possible implication of type I IFN still remains interesting because the IFN paradox was also observed in patients with cancer (*50*). Further explorations with a larger number of patients might be helpful to clearly exclude the implication of type I IFN.

In tandem with CMV seropositivity, a major inducer of senescence could be cancer-induced chronic antigenic stimulation. Many studies consistently report increased proportions or numbers of senescent circulating T cells in various type of cancer: head and neck (*51*), primary breast (*52*), ovarian (*53*), or lung (*54*). It is now well known that cancer could accelerate differentiation of T cells into a senescent state, and their treatments such as DNA-damaging chemotherapy could lead to premature aging of T cells with CD28 loss (*14*). In this study, we have shown that patients with advanced NSCLC have a higher proportion of senescent CD8^+^ T cells compared to patients with localized NSCLC and healthy volunteers. Our work also suggested a correlation between tumor burden and CD8^+^ T cell senescence. However, aNSCLC did not seem to act as a driver of T senescence itself because the proportions of CD8^+^ senescent T cells in CMV^−^ patients with IIIB-IV stages were similar to those of healthy volunteers, suggesting that even in the presence of cancer, CMV remains a mandatory driver of T cell senescence. The underlying mechanism for CMV-induced CD8^+^ T cell senescence in patients with aNSCLC remains to be properly explained.

This research is a first step toward a more profound understanding of CMV-induced lymphocyte senescence in patients likely to receive ICB, which could help in therapeutic decision-making. Only few studies have focused on the link between CMV infection and ICB clinical benefit. Some clinical cases have linked CMV reactivation to either ICB-induced colitis (*55*) or pneumonitis (*56*), but to our knowledge, this work is the first to identify CMV as a major viral driver among human-tropic viruses in T cell senescence in patients with cancer at least for NSCLC and RCC. Although CMV^+^ status showed a negative impact on PFS, this effect was no longer observed after adjustment on T_8_sen status, meaning that CMV^+^ impact is mainly driven by T_8_sen^high^ status. Moreover, this result was not found in PCT-treated patients, which means that T_8_sen^high^ status is predictive of resistance to ICB. These findings highlight the importance of host immune characteristics, specifically before the initiation of ICB treatment in patients with cancer. The negative impact of T_8_sen^high^ status on ICB response should be prospectively evaluated along with CMV serology and studied in other tumor types. Other studies have looked at markers of senescence or terminal differentiation of T cells before initiation of ICB treatment. In patients with advanced melanoma, a high baseline proportion of terminally differentiated effector memory CD8 was negatively associated with OS upon anti–Cytotoxic T-Lymphocyte-Associated protein-4 (CTLA-4) treatment (*57*). These results suggest the deleterious presence of circulating senescent or highly differentiated T cells in patients with different tumor types treated by ICB. Additional studies are needed to characterize the CMV-mediated mechanisms of senescent T cell accumulation in patients with cancer. The identification of molecular pathways should provide the basis for therapeutic strategies targeting the underlying mechanisms of senescence. More broadly, preventive approaches using anti-CMV vaccination could be envisaged to avoid the accumulation of senescent T cells in vulnerable patients.

## MATERIALS AND METHODS

### Experimental design

The samples and clinical data from 317 patients with NSCLC, 51 patients with RCC, and 31 healthy volunteers were used in this work. The T_8_sen status was first assessed in whole-blood samples from 306 patients with NSCLC and 31 healthy volunteers and in thawed PBMC from 51 patients with RCC. Then, we focused on 238 patients with aNSCLC, of which 228 were evaluable for T_8_sen status and 212 for CMV serology.

The characteristics of T_8_sen cells were first investigated by an intracellular staining of T-bet and Eomes on the PBMC of 15 patients with aNSCLC. A staining of SA-βgal along with a CD8^+^ T cell characterization was performed on the PBMC of seven patients with cancer.

We then investigated the factors associated with CD8^+^ T cell senescence in patients with aNSCLC. Among patients with aNSCLC having a T_8_sen status, a plasmatic dosage of some soluble factors and of oxidative stress biomarkers was performed on 79 and 94 samples, respectively. We measured the type I IFN signature using real-time quantitative PCR in the PBMC of 51 patients. On the basis of the rationale of an association of viral infections and immune cellular senescence, a VirScan assay allowed to screen for antibodies targeting human-tropic viruses in the serum of 176 patients with T_8_sen status.

Identification of CMV- or EBV-specific CD8^+^ T cells was assessed on thawed PBMC from 24 CMV^+^ and 20 EBV^+^ patients with aNSCLC, respectively. Identification of gB-specific CD4^+^ T cells was assessed on thawed PBMC from 10 patients with aNSCLC. Stimulation with gB peptide was performed on thawed PBMC from 11 patients with aNSCLC. Measurement of anti-CMV IgG titer was realized on 44 thawed plasma from samples of patients with aNSCLC.

After the identification of CMV as a biomarker for senescence in older patients, the clinical data of 212 patients with CMV serology was analyzed, of which 157 patients were treated with anti–PD-(L)1 monotherapy and had evaluable response in ICB discovery and validation cohorts and 42 in the chemotherapy cohort. Eleven patients in the PREMIS cohort were treated with a combination of chemotherapy and anti–PD-1 antibody and were not included in the survival analysis. A flow chart of the study design is provided in fig. S7.

### Patients, cohorts, and healthy volunteers

Healthy donor samples were collected from whole blood from the French Blood Establishment after donor agreement (EFS, agreement #18/EFS/021). PREMIS (NCT03984318, CSET 2018/2728, ICB-treated patients) is a biological study in which blood (plasma + serum + PBMC) of patients with advanced tumors was collected before the start of checkpoint blockade immunotherapy (alone or in combination with chemotherapy).

The CTC trial (NCT02666612, CSET 2008/1370, ICB-treated patients) is an interventional study in which blood was collected from adult patients with metastatic lung cancer before treatment with anti–PD-1 or anti–PD-L1 therapy.

PRINCEPS (NCT02994576, CSET 2016/2362) is a phase 2 study evaluating 1 cycle of neoadjuvant atezolizumab (anti–PD-L1 antibody) in patients with stage IA-IIIA NSCLC. In that cohort, PBMCs were prospectively collected at baseline.

In the SENLOAD cohort, PBMCs were collected in patients with unresectable stage III NSCLC before chemoradiation therapy followed by maintenance anti–PD-L1 (durvalumab) therapy.

In the MSN cohort (NCT02105168, PCT-treated patients), blood was prospectively collected from patients with stage IV melanoma or lung cancer to identify resistance mechanisms to anticancer treatments. In that cohort, patients with advanced NSCLC were treated with chemotherapy.

All patients provided informed consent. These studies were approved by the human research ethics committee of the institution and conducted in conformity with the International Conference on Harmonization and the Declaration of Helsinki.

### Blood collection, PBMC, plasma, and serum isolation

PBMCs were isolated on a Ficoll gradient from patients’ blood and cryopreserved in liquid nitrogen. Plasma and serum were extracted from blood in a heparinized tube and a dry tube, respectively, by centrifugation at 2800 rpm for 15 min at 15°C. A second centrifugation of the plasma was made (5000 rpm, 20 min, and 4°C) to remove residual platelets. The samples were stored at −80°C for later protein quantification.

### PBMC stimulation

A peptide derived from HCMV glycoprotein gB (217-227, DYSNTHSTRYV) was purchased from Bio-Synthesis (Lewisville, USA) and dissolved in dimethyl sulfoxide (DMSO). PBMCs were thawed, and 0.5 × 10^6^ cells were plated in 200 μl of RPMI 1640 supplemented with 10% fetal bovine serum (FBS) and 1% penicillin-streptomycin. Cells were incubated with gB-peptide (10 μg/ml) or an equivalent volume of DMSO for 20 hours at 37°C and 5% CO_2_. As a positive control, cells were incubated with phorbol 12-myristate 13-acetate (10 ng/ml) plus ionomycine (1 μg/ml) for 6 hours. For the final 5 hours of culture, brefeldin A (5 μg/ml; Sigma-Aldrich) was added to block cytokine secretion, and cells were collected for intracellular cytokine staining.

### Flow cytometry experiments

#### T-bet and Eomes assay

For each sample, 0.5 × 10^6^ thawed PBMCs in 50 μl of staining buffer [1× phosphate-buffered saline (PBS) supplemented with 0.4% EDTA, bovine serum albumin (BSA; 5 mg/ml), and sodium azide (1 mg/ml)] were incubated for 15 min at room temperature in the dark with antibodies targeting extracellular markers (“TF” panel; table S4), washed twice with staining buffer, and resuspended in 50 μl of FBS. T-bet and Eomes staining was performed using the PerFix-nc Kit (Beckman Coulter). Briefly, 5 μl of fixative reagent (R1) was added to the cells for 15 min at room temperature in the dark before adding the intracellular antibodies diluted in 300 μl of permeabilizing reagent (R2) for 1 hour at room temperature in the dark. After adding 3 ml of 1× R3 reagent, tubes were centrifuged for 5 min at 500*g*; cells were resuspended in 250 μl of 1× R3. Fluorescence was analyzed by flow cytometry using a Gallios flow cytometer (10 colors and three lasers; Beckman Coulter). Postacquisition analysis was done using the software Kaluza Analysis (Beckman Coulter).

#### SA-βgal assay

SA-βgal, detectable in lysosomes at pH 6.0, allows the identification of senescent cells. A total of 0.5 × 10^6^ freshly isolated PBMCs were seeded in 1 ml of complete medium (RPMI + GlutaMAX medium supplemented with 1% sodium/pyruvate, 1% non-essential amino acid (NEAA) medium, 1% penicillin-streptomycin, 1% Hepes buffer, and 10% FBS) plus 0.1% 2-mercaptoethanol into a 24-well flat bottom plate (Sigma-Aldrich, USA). Cells were treated with 100 nM alkalizing bafilomycin A1 (*M* = 622.83 g/mol; 0.16 mM in DMSO; Bafilomycin A1 Ready Made Solution; Sigma-Aldrich, SM L1661.1ML) and incubated for 1 hour at 37°C and 5% CO_2_. Then, the SA-βgal substrate 5-dodecanoylaminofluorescein di-β-d-galactopyranoside (C_12_FDG; *M* = 853.9156 g/mol; Invitrogen, D2893) is added at 33 μM, and cells were incubated in the dark for 1 hour at 37°C and 5% CO_2_. After harvesting, cells were washed twice with 3 ml of chilled 1× PBS and centrifuged at 300*g* for 5 min. The pellet was resuspended in 100 μl of 1× PBS, and cells were stained by extracellular antibodies (“SAβgal” panel; table S4) for 15 min at 4°C in the dark. Cells were then washed with 1× PBS and centrifuged for 5 min at 300*g*; the pellet was resuspended in 250 μl of 1×PBS, and tube was acquired on a CytoFLEX flow cytometer (Beckman Coulter). Expression of SA-βgal is given by the fluorescence of its hydrolyzed substrate C_12_FDG that emits green fluorescence at 520 nm.

#### Whole-blood immunophenotyping

Heparin tubes were used for fresh whole-blood immune phenotyping. For surface staining on blood, fresh whole blood (100 μl) was incubated for 20 min at room temperature in the dark with liquid antibodies (“T_8_sen” panel; table S4). Erythrocytes lysis was performed adding 1 ml of VersaLyse (Beckman) containing 25 μl of Fixative Solution (Beckman) for 20 min at room temperature in the dark. After two washings, cells were resuspended in 250 μl of 1× PBS. Stained cells were acquired using a Gallios flow cytometer (Beckman Coulter) and analyzed using Kaluza Analysis software (Beckman Coulter).

#### PBMC immunophenotyping

A total of 0.5 × 10^6^ thawed PBMCs were incubated with T_8_sen panel antibodies (table S4) for 15 min in the dark at 4°C. The cells were then washed with 3 ml of staining buffer and centrifuged at 300*g* for 5 min. The pellet was resuspended in 250 μl of staining buffer, and the fluorescence was analyzed by flow cytometry (Gallios, Beckman Coulter).

#### Determination of CMV- and EBV-specific CD8^+^ and CD4^+^ T cells

CMV-specific CD8^+^ T cells and EBV-specific CD8^+^ T cells were quantified by MHC I Dextramer following the manufacturer’s instructions (Immudex). PBMCs from HLA-A*0201 patients were thawed, and 0.5 × 10^6^ to 1 × 10^6^ cells were resuspended in 50 μl of wash buffer (PBS 1× + 5% FBS). Cells were incubated with two CMV MHC I Dextramer (VLEETSVML-IE1/PE, catalog no. WB02658 PE 50; NLVPMVATV-pp65/APC, catalog no. WB02132 AP 50) or two EBV MHC I Dextramer (LLDFVRFMGV-EBNA 3B/APC, catalog no. WB02143 AP 50; GLCTLVAML-BMLF1/PE, catalog no. WB02130 PE 50) for 10 min at room temperature in the dark. Surface antibody (“CD8 Dextramers” panel; table S4) panels were added, and cells were then incubated 20 min at room temperature in the dark. Cells were washed twice with 2 ml of wash buffer and centrifuged at 300*g* for 5 min. The pellet was resuspended, and fluorescence was analyzed on a flow cytometer (Gallios, Beckman Coulter). Dextramer-positive cells were identified among CD8^+^ T cells; the gate was set on the CD4^+^ cells that are negative for Dextramer staining.

CMV-specific CD4^+^ T cells were quantified using ProT2 MHC Class II Tetramer (ProImmune). A total of 1 × 10^6^ thawed PBMCs from DRB1*07:01 patients were resuspended in 50 μl of wash buffer (0.1% sodium azide and 0.1% BSA in PBS). Cells were stained with ProT2 MHC Class II Tetramer (PDDYSNTHSTRYVTV-gB/PE, TT2721-2A-E) and incubated for 2 hours at 37°C. Then, cells were washed once with wash buffer and stained with surface antibodies (“CD4 Tetramers” panel; table S4) and incubated on ice for 20 min in the dark. Cells were washed twice with 2 ml of wash buffer and centrifuged at 300*g* for 5 min. The pellet was resuspended, and fluorescence was analyzed on a flow cytometer (Gallios, Beckman Coulter). CD4 gB-specific cells were identified among CD4^+^ cells after excluding CD19^+^ cells as tetramers can bind nonspecifically to B cells. The gate was set on the CD8^+^ cells that are negative for Tetramer staining. Phenotyping of CD4 gB-specific was performed among samples with sufficient number of cells.

#### Intracellular cytokine staining

Stimulated PBMCs were washed and resuspended in 50 μl of FBS for intracellular cytokine staining using the PerFix-nc kit (Beckman Coulter) and antibodies of the “Cytokines” panel (table S4). Cells were previously stained by surface antibodies for 5 min at room temperature in the dark. Then, 2,5 μl of fixative reagent (R1) was added to the cells for 15 min at room temperature in the dark before adding the intracellular antibodies diluted in 150 μl of permeabilizing reagent (R2) for 1 hour at room temperature in the dark. After adding 3 ml of 1× R3 reagent, tubes were centrifuged for 5 min at 500*g*; cells were resuspended in 250 μl of 1× R3, and tubes were acquired on a Gallios flow cytometer (Beckman Coulter).

### Plasmatic dosages

#### CMV serology and anti-CMV IgG titer

CMV serology was performed on thawed plasma by a sandwich immunoassay (Atellica IM CMV IgG, Siemens) and by enzyme-linked immunosorbent assay (ELISA; Abcam anti-CMV IgG Human ELISA Kit, catalog no. ab108724) that allows qualitative and semiquantitative detection of IgG directed against CMV. Plasma and serum anti-CMV IgG titers were measured by ELISA (Abcam anti-CMV IgG Human ELISA Kit, catalog no. ab108724) using a two-step dilution protocol (1:400 to 1:51,200) and following the manufacturer’s instructions.

#### Detection and quantification of plasmatic proteins

All plasma samples were thawed and centrifuged at 5000 rpm for 20 min at 4°C to remove residual platelets. Cytokines and soluble proteins were measured using the Meso Scale Discovery (MSD) immunoassay (Rockville, MD, USA). Four MSD kits were used: “U-PLEX Immuno-Oncology Group 1 (hu)” to measure IFN-β, IL-6, IL-8, IL-10, IL-29/IFN-λ1, IP-10, PD-L1, and TNF-α; “S-PLEX Human IFN-α2a” to measure IFN-α2a; “V-PLEX Human VCAM-1” to measure VCAM-1; and “R-PLEX Human MPO” to measure myeloperoxydase (MPO). Assays were performed following the manufacturer’s protocol using MSD 96-well plates and recommended diluents. Plates were read using MESO QuickPlex SQ 120 (MSD LLC), and raw data were analyzed with the Discovery Workbench 4.0 software (MSD LLC). All measurements were run in duplicate for the calculation of coefficient of variation and means, allowing the elimination of means with a coefficient of variation (CV) > 25%. Quantitative measurement of human neutrophil elastase was performed by sandwich ELISA using the Human Neutrophil Elastase ELISA Kit (Abcam, ab270204) according to the manufacturer’s instructions. The lower limits of detection (LLOD) and sample dilutions are described in table S5.

### Real-time qPCR for gene expression analyses and type I IFN score

#### RNA extraction

Total RNA was isolated from a minimum of 2 × 10^6^ thawed PBMCs from patients and negative controls (healthy volunteers) by phase separation using TRI reagent (200 ml; #TR-118, Sigma-Aldrich) and chloroform (#22716.296, Prolabo). Then, RNA was precipitated with isopropanol (#4151154, Carlo Erba), washed with 70% ethanol (absolute ethanol; #414607, Carlo Erba) and ribonuclease-deoxyribonuclease (RNAse-DNAse)–free water (#20-104, Qbiogene), and redissolved in 20 μl of RNAse-DNAse–free water.

#### Reverse transcription

A maximum of 1 μg of RNA, measured by fluorometric kits (Qubit RNA HS Assay Kit, no. Q32855; Qubit RNA BR Assay Kit, no. Q10210) was reverse-transcribed into cDNA with a reaction mix of the cDNA Synthesis Kit (Invitrogen, SuperScript Vilo cDNA Synthesis Kit, no. 11754050).

#### Real-time PCR reaction for gene expression

Expression of *MX1* (Hs00895608_m1), *IFITM1* (Hs00705137_s1), *IFIT1* (Hs01675197_m1), *IFI44* (Hs00197427_m1), *LY6E* (Hs00158942_m1), and housekeeping gene *ACTB* (Hs99999903_m1; all from Thermo Fisher Scientific; table S6) was analyzed in 10 ng of cDNA with the TaqMan Fast Advanced Master Mix (Applied Biosystems) containing polymerase buffer with each TaqMan assay as recommended from the provider. Amplifications were carried out with thermocycler ViiA 7 (Thermo Fisher Scientific).

#### Calculation of relative expression

Values for each transcript were normalized to expression of the housekeeping gene *ACTB*. Relative expressions were determined from normalized CT values (CT gene–CT housekeeping gene) using the comparative CT (2^−ΔΔCT^) by standardizing the expression with mean and SD of each gene in the negative control group.

#### Type I IFN score

Type I IFN score for each patient was defined by the sum of the relative expression of five ISGs: *MX1*, *IFITM1*, *IFIT1*, *IFI44*, and *LY6E*. Sums superior to 10 represent type I IFN score positivity (*58*).

### CMV viral load

Quantitative real-time PCR was used for measuring CMV DNA amplification. DNA extraction was performed automatically on the QIAsymphony SP platform (QIAGEN) using the DSP DNA Midi Kit from patient’s whole-blood samples. Detection and quantification of CMV DNA was performed on 30 μl of DNA elute volume using the CMV ELITe MGB Kit (ELITech Group, Italy) on the ELITe InGenius system.

### VirScan phage display immunoprecipitation and sequencing

#### Study population

The sera of 176 patients with NSCLC treated with ICB (PREMIS study, *n* = 115) and with PCT (MSN study, *n* = 61) with a T_8_sen status were selected for the VirScan assay (CDI Laboratories).

#### VirScan assay

This assay, which uses phage display immunoprecipitation and sequencing, is a sensitive and focused high-comprehensive approach that enables thorough serological profiling of antiviral antibodies in humans and, consequently, the identification of viral exposure throughout the human virome. The process for this method has been extensively explained elsewhere (*59*), and it has undergone extensive reliability and validity evaluations. Briefly, all proteins from 206 human-tropic viruses (representing more than 1000 strains) in the UniProt library were divided into 106678 56mer-peptide tiles with 28–amino acid overlap. This oligonucleotide library (VirScan, Version Vir3, ~110,000 viral peptides) (*60*) was then PCR-amplified with adaptors for cloning and inserted in a T7 phage display vector, which was expanded in *Escherichia coli*. The samples were then incubated overnight at 4°C with this phage library. The antibodies and the phages to which they are bound are then immunoprecipitated using protein A/G–coated magnetic beads. The unbound phages are removed by washing. The same procedure is performed on a negative control reagent containing no antibody. The DNA sequences of bound phages are then amplified by PCR. A second hemi-nested PCR allows the addition of sample-specific barcodes. DNA is then sequenced at high throughput to quantify the enrichment of each phage and, indirectly, the presence and quantity of antibody targeting a specific peptide.

#### Processing of raw data

For each patient, the phage abundance for each viral peptides is reported in fold change, comparing the number of each oligonucleotide, i.e., viral peptide, in the sample to a control reagent containing no antibody. Reactivity of a patient’s serum toward a viral peptide can be defined by a fold change strictly greater than 1, and we considered that reactivity against a viral protein was positive if the patient had at least one positive antipeptide antibody.

For the unsupervised analysis, each peptide is plotted depending on the log_2_ fold change in the mean percent T_8_sen among CD8^+^ T cells between patients positive and negative for this peptide [threshold for significance: abs(log_2_ fold change) > 0.4854 i.e., 40% change in the mean %T_8_sen concentration] and on the corresponding *P* value [−log_10_ base, threshold for significance: −log_10_(*P* value) > 2, i.e., *P* < 0.01].

### Statistical analysis

Continuous variables were described by their median and compared by a Student or Mann Whitney test, when necessary. Associations between continuous variables were assessed using a Spearman test. Categorical variables are presented as percentages and compared using a chi-square or Fisher test when necessary.

PFS was defined as the time from the start of treatment to the first scan showing progression. OS is defined as the time from initiation of treatment to death. Survivals were expressed as medians with their 95% CIs using the Kaplan-Meier method. Survival curves were compared by a log rank test. The association between the different variables of interest and the survival criteria (OS and PFS) were studied using a univariate Cox model.

For each test, a difference was considered significant if the first-species risk was less than 5% (*P* < 0.05). Statistical analyses and figures were performed using R software. The specific statistical pipeline analysis for VirScan assay is described above.
