# Supplementary material for: Human virome profiling identified CMV as the major viral driver of a high accumulation of senescent CD8+ T cells in patients with advanced NSCLC
Source: Sci Adv. 2023 Nov 8;9(45):eadh0708. doi: 10.1126/sciadv.adh0708 (PMC10631735; doi:10.1126/sciadv.adh0708)
Supplement: Supplementary file 1 — Figs. S1 to S7 Tables S1 to S6 [file sciadv.adh0708_sm.pdf]

Science Advances
AAAS

Supplementary Materials for

Human virome profiling identified CMV as the major viral driver of a high accumulation of senescent CD8$^+$ T cells in patients with advanced NSCLC

Marie Naigeon *et al.*

Corresponding author: Nathalie Chaput, nathalie.chaput@gustaveroussy.fr

Sci. Adv. **9**, eadh0708 (2023)
DOI: 10.1126/sciadv.adh0708

This PDF file includes:

Figs. S1 to S7
Tables S1 to S6

Fig. S1. CD28^{-}CD57^{+}KLRG1^{+}CD8^{+} circulating T cells are characterized by higher expression of the transcription factor T-bet and SA-βgal (A) MFI of SA-βgal was determined in fresh PBMC from n=7 patients with aNSCLC and subsequently analyzed among CD8^{+} T cell subpopulations. **(B)** The expression of T-bet and Eomes transcription factors was assessed on thawed PBMC (n=15) from aNSCLC patients, and visualized among T cell CD8^{+} populations as shown on the gating strategy. **(C-D)** Eomes and T-bet expression was analyzed among CD8^{+} subpopulations according to the expression of senescent markers. Differences between groups were analyzed by Wilcoxon test.

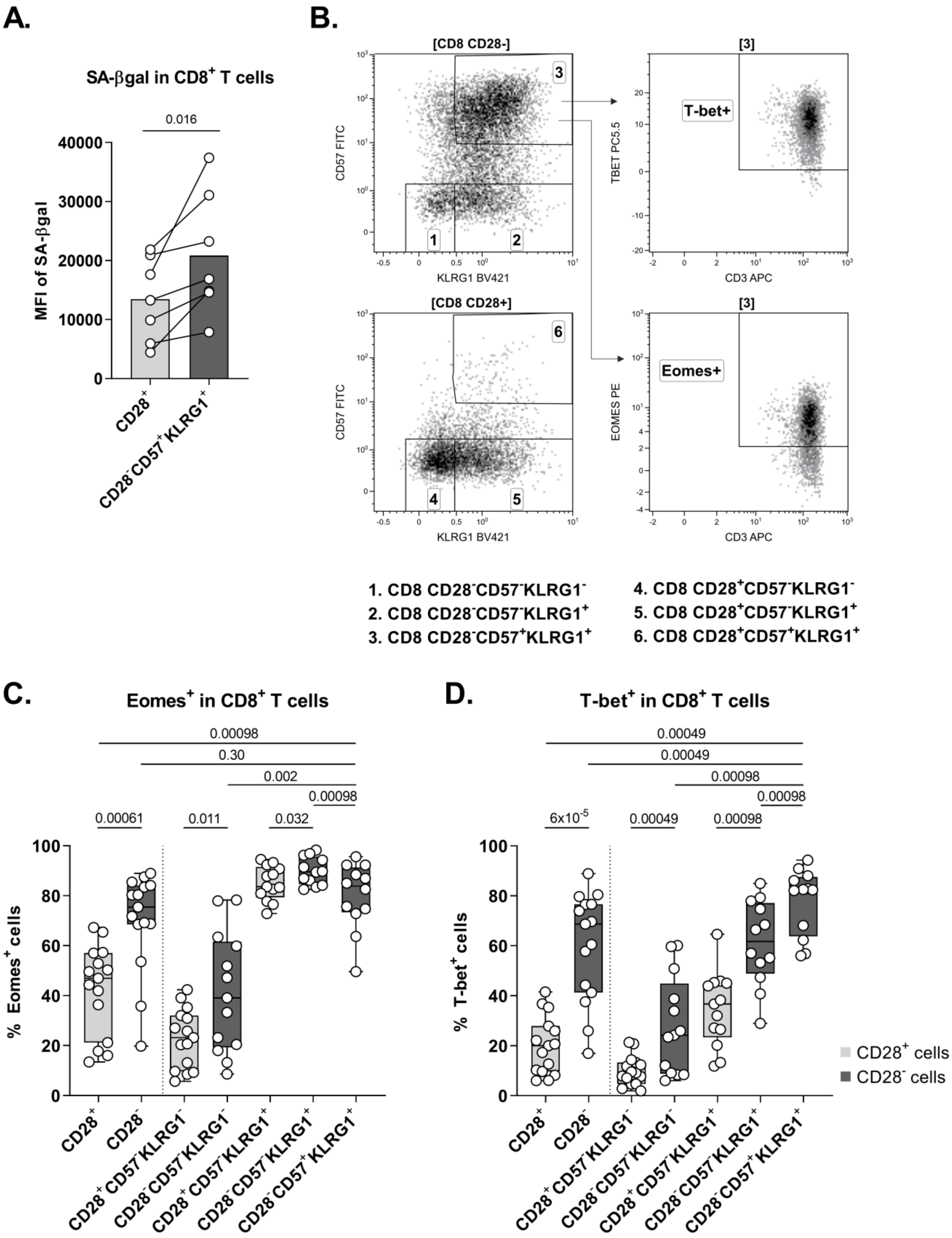

A.
SA-βgal in CD8+ T cells
0.016
MFI of SA-βgal
40000
30000
20000
10000
0
CD28+
CD28-CD57+KLRG1+
B.
[CD8 CD28-]
CD57 FITC
KLRG1 BV421
3
1
2
[3]
T-bet+
TBET PC5.5
CD3 APC
[CD8 CD28+]
6
4
5
Eomes+
EOMES PE
1. CD8 CD28-CD57-KLRG1-
2. CD8 CD28-CD57-KLRG1+
3. CD8 CD28-CD57+KLRG1+
4. CD8 CD28+CD57-KLRG1-
5. CD8 CD28+CD57-KLRG1+
6. CD8 CD28+CD57+KLRG1+
C.
Eomes+ in CD8+ T cells
0.00098
0.30
0.002
0.00098
0.00061
0.011
0.032
% Eomes+ cells
100
80
60
40
20
0
CD28+
CD28-
CD28+CD57-KLRG1-
CD28-CD57-KLRG1-
CD28+CD57-KLRG1+
CD28-CD57-KLRG1+
CD28-CD57+KLRG1+
D.
T-bet+ in CD8+ T cells
0.00049
0.00049
0.00098
0.00098
6x10-5
0.00049
0.00098
% T-bet+ cells
100
80
60
40
20
0
CD28+
CD28-
CD28+CD57-KLRG1-
CD28-CD57-KLRG1-
CD28+CD57-KLRG1+
CD28-CD57-KLRG1+
CD28-CD57+KLRG1+
CD28+ cells
CD28- cells

Fig. S2. Comparison of VirScan™ assay and CMV serology. CMV-serology was assessed in plasma from samples with a VIRSCAN analysis (n=116). ROC curve comparing both methods showed a threshold of 4 recognized viral-proteins for viral-status positivity, with great sensibility and specificity.

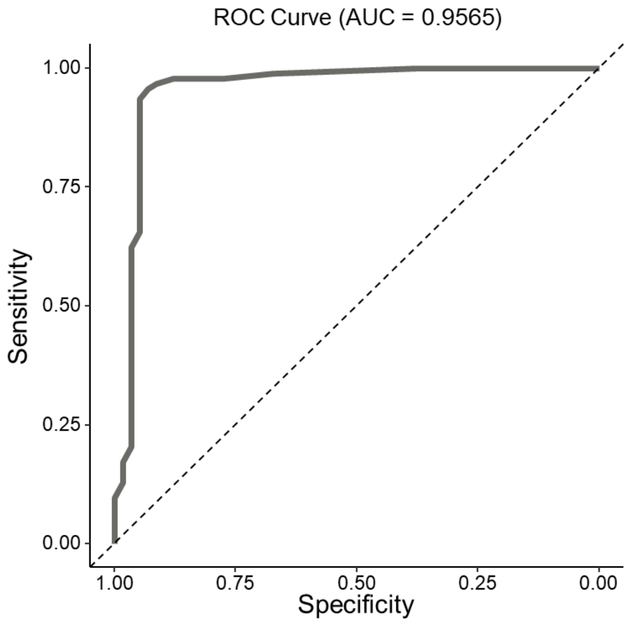

Fig. S3. **T_8sen-high status is associated with CMV+ status in RCC patients**. T_8sen status was assessed in thawed PBMC of 51 RCC patients with available CMV status. (A) Proportions of CMV+ patients are represented according to T_8sen status, proportions of T_8sen^{high} patients are represented according to CMV status. (B) %T_8sen is compared between CMV+ and CMV- patients. The horizontal dotted line represent a proportion of $CD8^+$ senescent T cells of 39.5% among $CD8^+$ cells. Categorical variables ($T_8sen^{high/low}$, CMV+/-) were compared by Fisher test, continuous variables were compared in two populations by Mann-Whitney t-test.

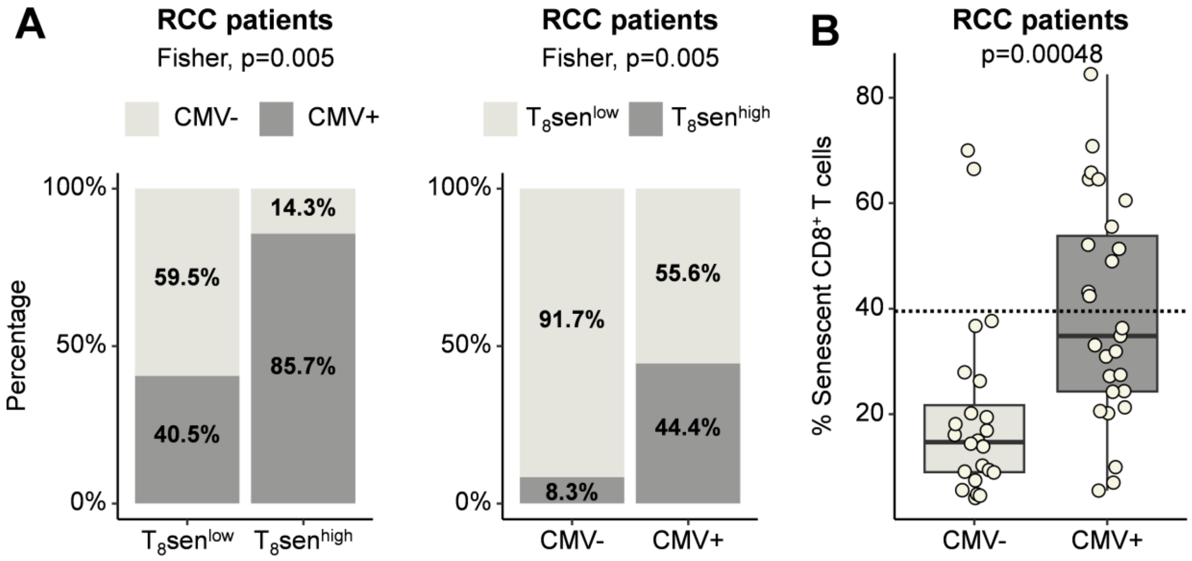

Fig. S4. Impact of coinfections with other herpesviruses in CMV+ patients. Proportions of $CD8^+$ senescent T cells among $CD8^+$ cells is compared between seropositive and seronegative patients for other herpesviruses in n=117 CMV+ patients (VirScan). (B) %T_8sen depending on EBV and CMV coinfection status.

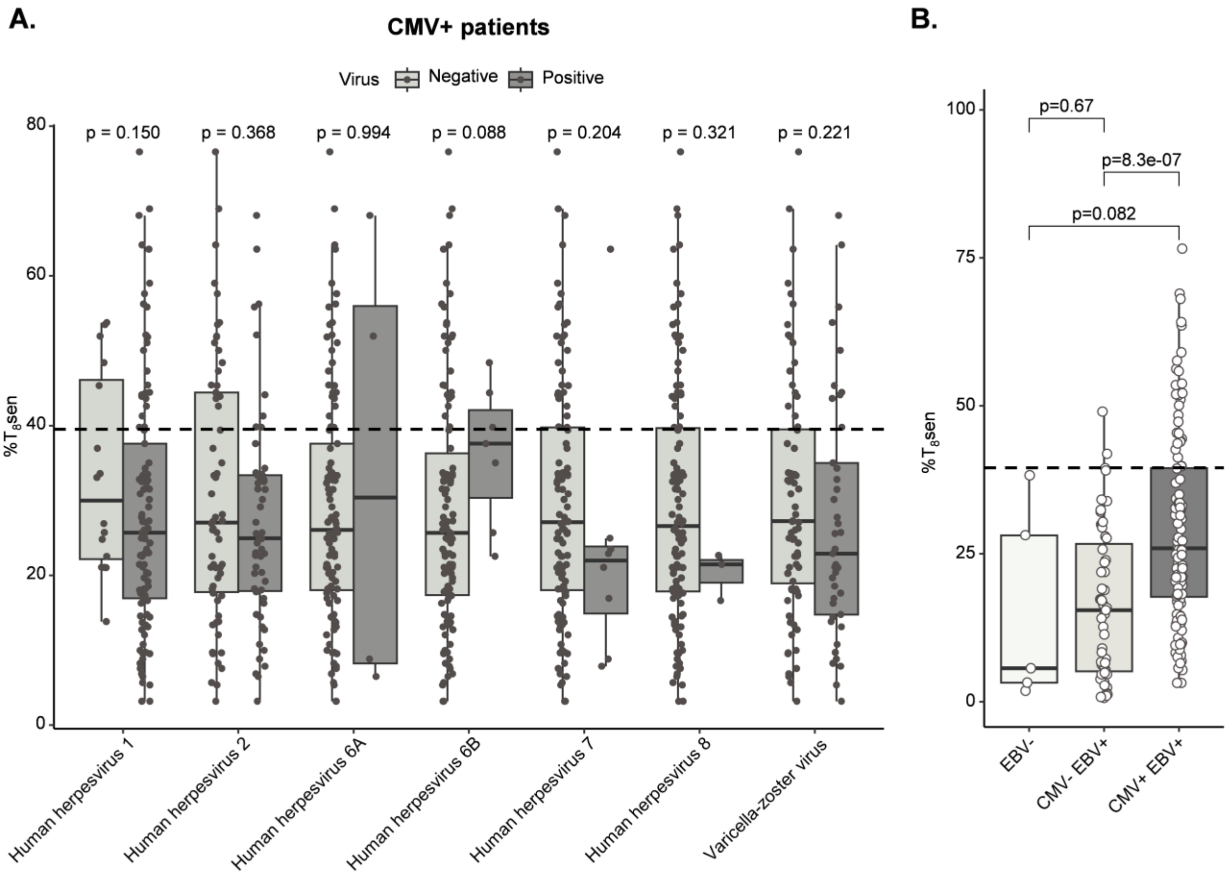

Fig. S5. Gating strategy for the identification of CMV and EBV-specific cells. Identification of (**A**) CMV (pp65 and IE1) and EBV (BMLF1 and EBNA 3B) specific $CD8^+$ cells ; (**B**) CMV (gB) specific $CD4^+$ cells ; (**C**) IFN-γ^+-producing $CD4^+$ cells after stimulation with DMSO, PMA/ionomycin or gB peptide.

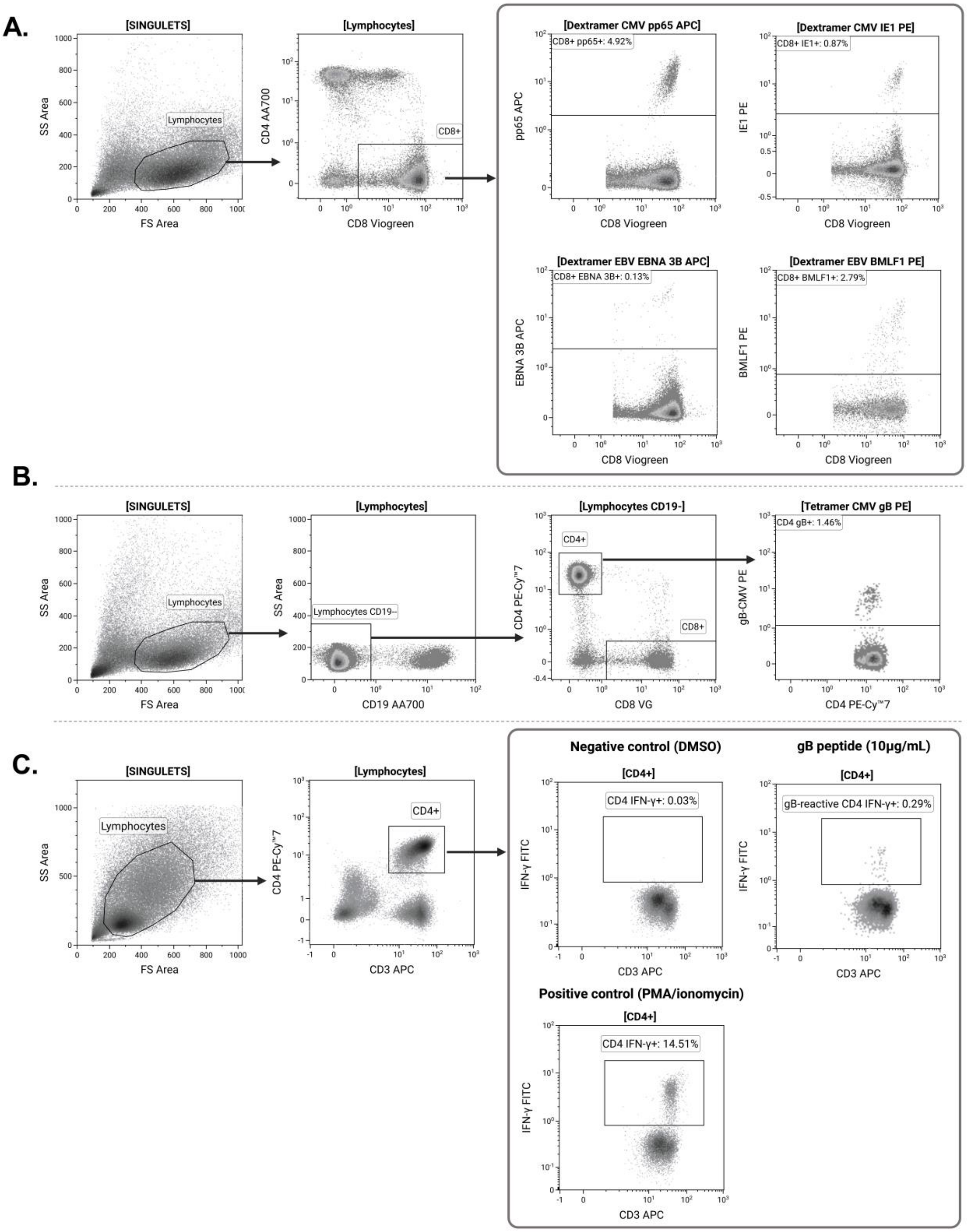

A.
[SINGULETS]
Lymphocytes
SS Area
FS Area
[Lymphocytes]
CD8+
CD4 AA700
CD8 Viogreen
[Dextramer CMV pp65 APC]
CD8+ pp65+: 4.92%
pp65 APC
[Dextramer CMV IE1 PE]
CD8+ IE1+: 0.87%
IE1 PE
[Dextramer EBV EBNA 3B APC]
CD8+ EBNA 3B+: 0.13%
EBNA 3B APC
[Dextramer EBV BMLF1 PE]
CD8+ BMLF1+: 2.79%
BMLF1 PE
B.
[SINGULETS]
Lymphocytes
[Lymphocytes]
Lymphocytes CD19–
CD19 AA700
[Lymphocytes CD19-]
CD4+
CD8+
CD4 PE-Cy™7
CD8 VG
[Tetramer CMV gB PE]
CD4 gB+: 1.46%
gB-CMV PE
C.
[SINGULETS]
Lymphocytes
[Lymphocytes]
CD4+
CD3 APC
Negative control (DMSO)
[CD4+]
CD4 IFN-γ+: 0.03%
IFN-γ FITC
gB peptide (10µg/mL)
[CD4+]
gB-reactive CD4 IFN-γ+: 0.29%
Positive control (PMA/ionomycin)
[CD4+]
CD4 IFN-γ+: 14.51%

Fig. S6. CMV and T_8sen status were not associated with PFS nor OS in the PCT-treated cohort. In 42 aNSCLC patients treated by PCT, **(A)** probability of PFS and OS depending on CMV status and **(B)** PFS and OS depending on CMV and T_8sen status. Survival curves were analyzed using a Cox regression model.

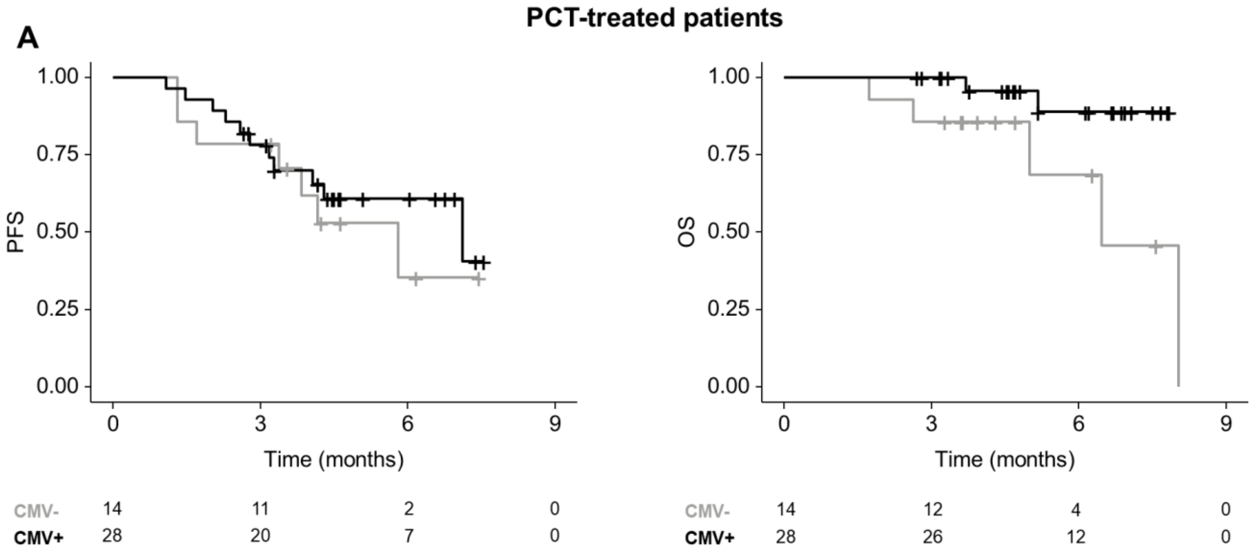

Groups	HR (%95 CI)	*p*
**CMV+** vs. CMV-	0.73 (0.28-1.89)	*0.52*

Groups	HR (%95 CI)	*p*
**CMV+** vs. CMV-	0.19 (0.034-1.02)	*0.053*

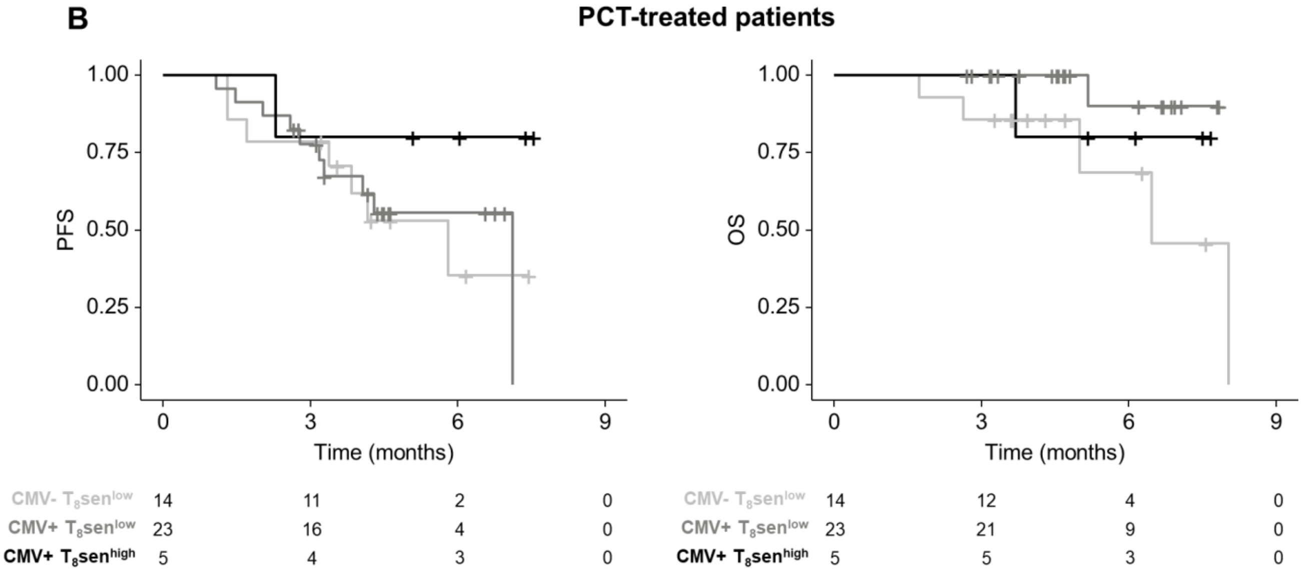

Groups	HR (%95 CI)	*p*
CMV- T_8sen^{low} vs. CMV+ T_8sen^{low}	0.90 (0.34-2.37)	*0.83*
**CMV+ T_8sen^{high}** vs. CMV+ T_8sen^{low}	0.25 (0.03-2.07)	*0.20*
**CMV+ T_8sen^{high}** vs CMV- T_8sen^{low}	0.27 (0.03-2.26)	*0.22*

Groups	HR (%95 CI)	*p*
CMV- T_8sen^{low} vs. CMV+ T_8sen^{low}	0.12 (0.01-1.09)	*0.059*
**CMV+ T_8sen^{high}** vs. CMV+ T_8sen^{low}	0.41 (0.05-3.75)	*0.43*
**CMV+ T_8sen^{high}** vs CMV- T_8sen^{low}	3.00 (0.19-48.5)	*0.44*

Fig. S7. Flow-chart of the study design. This figure illustrates the analyses performed on the different samples. Samples from patients assessed for **VirScan™**, type I IFN signature and plasmatic dosages had a SIP status. aNSCLC: advanced non-small cell lung cancer; CMV: cytomegalovirus; HV: healthy volunteers; ICB: immune-checkpoint blockers; IFN: interferon; MSD: meso-scale discovery quantification of soluble proteins; pts: patients; SA-βgal: senescence-associated beta-galactosidase; T_8sen: senescent $CD8^+$ T cells defined by the proportion of $CD28^-$ $CD57^+KLRG1^+$ T cells among $CD8^+$.

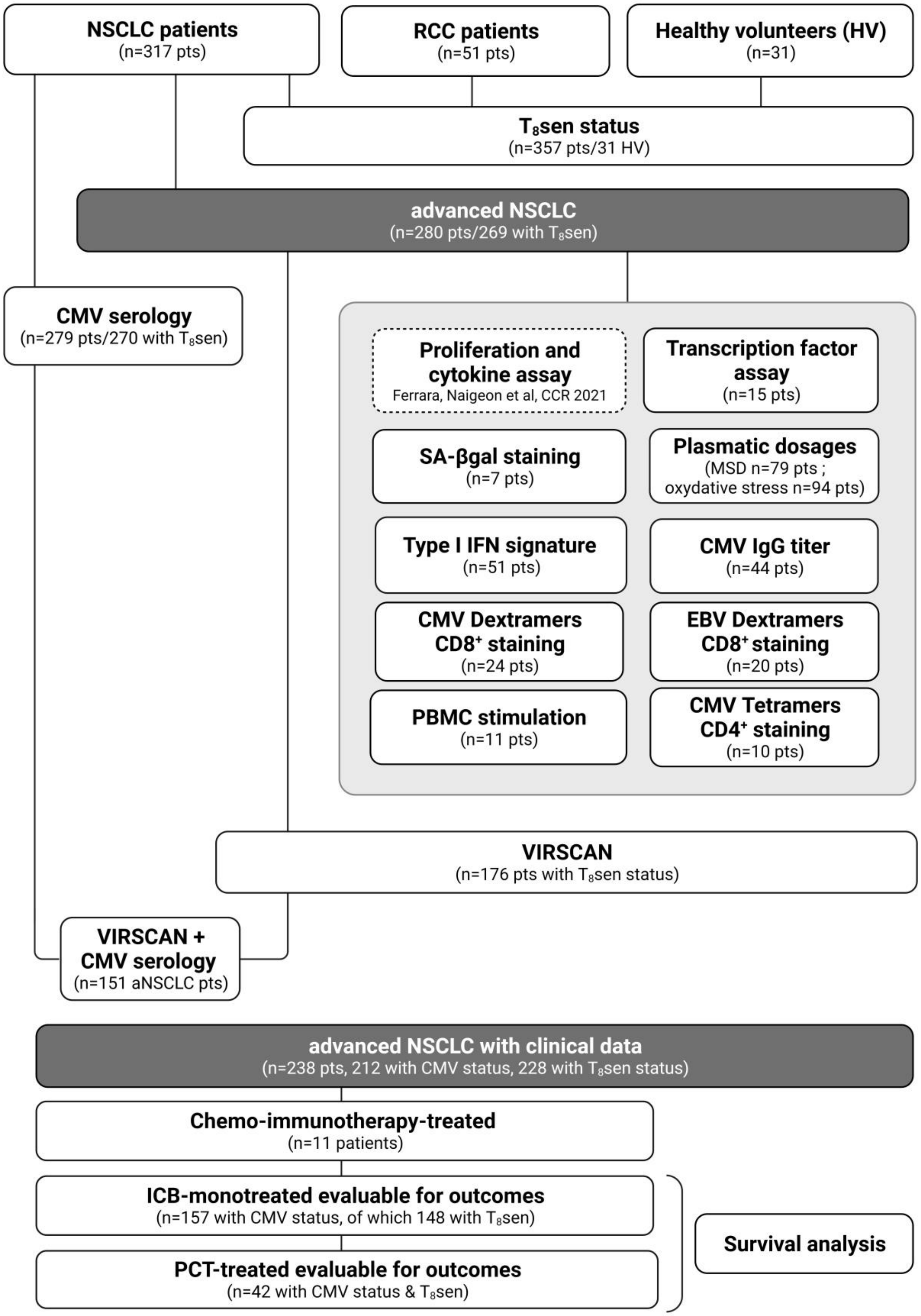
NSCLC patients
(n=317 pts)
RCC patients
(n=51 pts)
Healthy volunteers (HV)
(n=31)
T_8sen status
(n=357 pts/31 HV)
advanced NSCLC
(n=280 pts/269 with T_8sen)
CMV serology
(n=279 pts/270 with T_8sen)
Proliferation and cytokine assay
Ferrara, Naigeon et al, CCR 2021
Transcription factor assay
(n=15 pts)
SA-βgal staining
(n=7 pts)
Plasmatic dosages
(MSD n=79 pts ; oxydative stress n=94 pts)
Type I IFN signature
(n=51 pts)
CMV IgG titer
(n=44 pts)
CMV Dextramers $CD8^+$ staining
(n=24 pts)
EBV Dextramers $CD8^+$ staining
(n=20 pts)
PBMC stimulation
(n=11 pts)
CMV Tetramers $CD4^+$ staining
(n=10 pts)
VIRSCAN
(n=176 pts with T_8sen status)
VIRSCAN + CMV serology
(n=151 aNSCLC pts)
advanced NSCLC with clinical data
(n=238 pts, 212 with CMV status, 228 with T_8sen status)
Chemo-immunotherapy-treated
(n=11 patients)
ICB-monotreated evaluable for outcomes
(n=157 with CMV status, of which 148 with T_8sen)
PCT-treated evaluable for outcomes
(n=42 with CMV status & T_8sen)
Survival analysis

Table S1. aNSCLC patients' characteristics. Abbreviation: BMI, body mass index; CMV, cytomegalovirus; dNLR, derived neutrophils-to-lymphocyte ratio; CRP, C-reactive protein; ICB, immune checkpoint blockers; NA, not available; NR, not reached; OS, overall survival; PCT, polychemotherapy; PD-L1 TPS, PD-L1 tumor-proportion score; PFS, progression-free survival; PY, pack-year; T_8sen, senescent $CD8^+$ T cells defined by the proportion of $CD28^-CD57^+KLRG1^+$ T cells among $CD8^+$.

	PCT cohort - (n=61)	ICB-treated patients (PREMIS) - (n=121)	ICB-treated patients (CTC) - (n=56)
**Clinical characteristics**			
Age - median (range)	63 (38-92)	63.9 (31.5-87.2)	61.76 (39-92.86)
BMI - median (range)	NA	23.1 (16.2-37)	23.21 (15.22-31.95)
Smoking status			
Ongoing - n(%)	0 (0)	25 (20.66)	19 (33.93)
Stopped - n(%)	0 (0)	82 (67.77)	31 (55.36)
Never - n(%)	0 (0)	11 (9.09)	4 (7.14)
NA - n(%)	61 (100)	3 (0.98)	2 (3.57)
PY - median (range)	NA	34 (5-730)	40 (0-80)
**Tumor characteristics**			
Stage			
Stage IV - n(%)	51 (83.61)	114 (94.21)	47 (83.93)
Stage III - n(%)	10 (16.39)	7 (5.79)	7 (12.50)
NA - n(%)	0 (0)	0 (0)	2 (3.57)
PD-L1 TPS			
PD-L1 <1% - n(%)	0 (0)	35 (28.93)	13 (23.21)
PD-L1 1-49% - n(%)	0 (0)	34 (28.1)	9 (16.07)
PD-L1 ≥50% - n(%)	0 (0)	43 (35.54)	17 (30.36)
NA - n(%)	61 (100)	9 (7.44)	17 (30.36)
Number of metastatic sites - median (range)	1 (0-7)	3 (1-6)	2 (0-5)
Molecular biology			
KRAS mutation - n(%)	15 (24.59)	44 (36.36)	22 (39.29)
EGFR mutation - n(%)	1 (1.64)	5 (4.13)	0 (0)
ALK alteration - n(%)	1 (1.64)	0 (0)	0 (0)
Other targetable alteration - n(%)	23 (37.7)	3 (2.48)	7 (12.5)
No targetable alteration - n(%)	19 (31.15)	23 (19.01)	16 (28.57)
NA - n(%)	2 (3.28)	46 (38.02)	11 (19.64)
Radiotherapy before or after immunotherapy			
Yes - n (%)	0 (0)	48 (39.67)	29 (51.79)
No - n (%)	0 (0)	47 (38.84)	25 (44.64)
NA - n (%)	61 (100)	26 (21.49)	2 (3.57)
Previous chemotherapy			
Yes - n (%)	0 (0)	61 (50.41)	49 (87.50)
No - n (%)	0 (0)	34 (28.1)	5 (8.93)
NA - n (%)	61 (100)	26 (21.49)	2 (3.57)
Immunotherapy line			
First line - n (%)	0 (0)	56 (46.28)	5 (8.93)
Second line - n (%)	0 (0)	50 (41.32)	40 (71.43)
Third line and more - n (%)	0 (0)	15 (12.4)	9 (16.07)
NA - n(%)	0 (0)	0 (0)	2 (3.57)
**Biology**			
T_8sen status (> or <39.5%)			
High (>39.5%) - n (%)	7 (11.48)	25 (20.66)	13 (23.21)

Low (≤39.5%) - n (%)	54 (88.52)	95 (78.51)	34 (60.71)
NA - n (%)	0 (0)	1 (0.83)	9 (16.07)
% T_8sen among CD8+ T cells - median (range)	22.54 (0.8-76.54)	22.23 (0-68.94)	22.11 (2.26-63.74)
CMV serology			
Positive - n (%)	28 (45.9)	67 (55.37)	30 (53.57)
Negative - n (%)	14 (22.95)	48 (39.67)	25 (44.64)
NA - n (%)	19 (31.15)	6 (4.96)	1 (1.79)
dNLR - median (range)	NA	3.23 (0.71-23.17)	2.32 (0.56-20.25)
Albumin - median (range)	NA	39 (23-49)	NA
CRP - median (range)	NA	21 (0-236)	NA
**Outcomes**			
PFS (months) - median (95%CI)	4.98 (4.16-7.11)	3.78 (2.76-5.75)	5.36 (2.01-7.99)
OS (months) - median (95%CI)	8.03 (7.07-NR)	12.89 (9.76-19.59)	13.18 (7.04-24.23)

Table S2. Number of patients with detectable antibodies targeting each virus screened with VirScan™ assay. Only viruses against which at least one patient was seropositive (n=48) are indicated

Virus	Number (%) of seropositive patients
Epstein-Barr virus	171 (97.16%)
Human respiratory syncytial virus	164 (93.18%)
Human herpesvirus 1	136 (77.27%)
Human cytomegalovirus	117 (66.48%)
Influenza A virus	93 (52.84%)
Poliovirus type 1	93 (52.84%)
Human herpesvirus 2	71 (40.34%)
Varicella-zoster virus	63 (35.8%)
Human adenovirus C serotype 2	59 (33.52%)
Influenza B virus	56 (31.82%)
Cercopithecine herpesvirus 16	33 (18.75%)
Human rhinovirus 23	27 (15.34%)
Cercopithecine herpesvirus 1	18 (10.23%)
Hepatitis B virus	14 (7.95%)
Influenza C virus	12 (6.82%)
Human herpesvirus 6B	10 (5.68%)
Human herpesvirus 7	9 (5.11%)
Rubella virus	9 (5.11%)
Human adenovirus A serotype 12	8 (4.55%)
Human herpesvirus 6A	8 (4.55%)
Chikungunya virus	7 (3.98%)
Human parvovirus B19	7 (3.98%)
Vaccinia virus	7 (3.98%)
Human adenovirus E serotype 4	5 (2.84%)
Human hepatitis A virus genotype IA	4 (2.27%)
Human herpesvirus 8	4 (2.27%)
Human respiratory syncytial virus A	4 (2.27%)
Orf virus	4 (2.27%)
Saimiriine herpesvirus 2	4 (2.27%)
Human adenovirus C serotype 5	3 (1.7%)
Human respiratory syncytial virus B	3 (1.7%)
Cowpox virus	2 (1.14%)
Hepatitis E virus	2 (1.14%)
Human adenovirus B serotype 7	2 (1.14%)
Human herpesvirus 8 type P	2 (1.14%)
Tanapox virus	2 (1.14%)
Yaba monkey tumor virus	2 (1.14%)
Zaire ebolavirus	2 (1.14%)
BK polyomavirus	1 (0.57%)
Hepatitis delta virus	1 (0.57%)
Human adenovirus 55	1 (0.57%)
Human adenovirus A serotype 18	1 (0.57%)
Human adenovirus B serotype 3	1 (0.57%)
Human adenovirus D serotype 9	1 (0.57%)
Human adenovirus F serotype 40	1 (0.57%)
Human coronavirus HKU1	1 (0.57%)
Human herpesvirus 3	1 (0.57%)

Human parainfluenza 3 virus	1 (0.57%)
Merkel cell polyomavirus	1 (0.57%)
Molluscum contagiosum virus subtype 1	1 (0.57%)
Reston ebolavirus	1 (0.57%)
Simian virus 40	1 (0.57%)
Torque teno virus	1 (0.57%)

Table S3. T_8sen^{high} status is not associated with specific EBV-protein immunization. Pan-virus serological profile was assessed in sera of 176 patients with aNSCLC. Protein reactivity was positive if at least one peptide was recognized (enrichment fold-change>1). In EBV+ patients (n=171), proportions of $CD8^+$ senescent T cells were analyzed according to reactivity against 22 proteins with at least 20% of protein-seropositive patients and 20% of protein-seronegative patients, by Mann-Whitney test. Proportions of protein-seropositive patients were then compared between T_8sen^{high} and T_8sen^{low} patients by Fisher's test. T_8sen: senescent $CD8^+$ T cells defined by the proportion of $CD28^-CD57^+KLRG1^+$ T cells among $CD8^+$; EBNA: EBV nuclear antigen

Protein	Number and [proportion] of protein-seropositive patients	Median $CD8^+$ senescent T cells proportions			Proportions of protein-seropositive patients		
		Seronegative patients	Seropositive patients	p-value	In T_8sen^{low} patients	In T_8sen^{high} patients	p-value
Nuclear antigen EBNA-1 (Fragment)	106 [61.99 %]	27.08	19.26	**0.0073**	65.00	48.39	0.1028
Envelope glycoprotein M (gM)	38 [22.22 %]	24.87	19.73	**0.0203**	25.00	9.68	0.0925
Putative uncharacterized protein	41 [23.98 %]	24.56	16.62	0.0699	25.00	19.35	0.6438
Deneddylase (EC 3.4.19.12/ EC 3.4.22.-) (Large tegument protein)	77 [45.03 %]	21.07	24.87	0.0886	42.86	54.84	0.2382
Capsid scaffolding protein (Capsid protein P40/pPR/BVRF2) [Cleaved into: Assemblin, Assembly protein]	99 [57.89 %]	23.10	22.53	0.0920	60.71	45.16	0.1586
EBNA-3 (EBNA-3A)	107 [62.57 %]	25.51	22.53	0.1425	65.71	48.39	0.0998
Envelope glycoprotein B (gB) (GP115) (Glycoprotein GP110)	43 [25.15 %]	23.22	22.53	0.1508	27.14	16.13	0.2558
BZLF1	119 [69.59 %]	24.90	21.92	0.2032	70.00	67.74	0.8308
BZLF2 (Fragment)	47 [27.49 %]	23.10	22.54	0.2436	29.29	19.35	0.3738
Glycoprotein 42 (gp42)	106 [61.99 %]	24.96	22.21	0.3231	62.14	61.29	1.0000
Uncharacterized protein BLRF3 (Fragment)	36 [21.05 %]	24.31	18.25	0.3321	20.71	22.58	0.8103
Trans-activator protein BZLF1 (EB1) (Zebra)	115 [67.25 %]	23.22	22.53	0.3357	67.86	64.52	0.8328
BLLF1	79 [46.2 %]	25.20	21.42	0.3502	47.14	41.94	0.6919
EBNA-4 (EBNA-3B)	126 [73.68 %]	24.81	22.54	0.4078	75.00	67.74	0.4989
EBNA-LP (EBNA-5)	78 [45.61 %]	23.90	21.66	0.4094	47.86	35.48	0.2367

Envelope glycoprotein GP350 (Membrane antigen)	85 [49.71 %]	24.89	21.42	0.4735	50.71	45.16	0.6920
Tegument protein BKRF4	50 [29.24 %]	21.89	24.11	0.6333	28.57	32.26	0.6685
Tegument protein BLRF2	123 [71.93 %]	23.22	22.53	0.7479	70.71	77.42	0.5148
EBNA2 (Fragment)	131 [76.61 %]	24.80	22.54	0.7773	78.57	67.74	0.2407
EBNA1 (Fragment)	133 [77.78 %]	25.27	21.92	0.8541	76.43	83.87	0.4769
EBNA-1 protein (Fragment)	77 [45.03 %]	23.51	21.89	0.8852	44.29	48.39	0.6946
EBNA3B	95 [55.56 %]	22.73	22.65	1.0000	54.29	61.29	0.5516

Table S4. Antibodies used in flow cytometry experiments.
AA: allophycocyaning Alexa Fluor; APC: allophycocyanin; BV: Brilliant Violet™; ECD: electron-coupled dye; FITC: fluorescein isothiocyanate; KrOr: krome orange; PC5.5: PerCP-cyanine 5.5; PCy7: phycoerythrin-cyanine 7; PE: phycoerythrin.

Target	Fluorochrome	Provider	Dilution	Clone	Reference	Panel
CD28	APC-Vio770	Miltenyi Biotec	1:50	REA612	130-116-506	TF, T_8sen
CD28	ECD	Beckman Coulter	1:10	CD28.2	6607111	SA-βgal, T_8sen, CD8 Dextramers, CD4 Tetramers
CD28	PE-Vio615	Miltenyi Biotec	1:50	REA612	130-120-034	T_8sen
CD28	PE	Beckman Coulter	1:10	CD28.2	IM2071U	T_8sen
CD3	APC	Miltenyi Biotec	1:50	REA613	130-113-135	TF, Cytokines
CD3	AA750	Beckman Coulter	1:20	UCHT1	A94680	SA-βgal, T_8sen
CD3	APC-Vio770	Miltenyi Biotec	1:50	REA613	130-113-136	T_8sen
CD3	PE-Vio615	Miltenyi Biotec	1:50	REA613	130-114-520	T_8sen
CD4	AA700	Beckman Coulter	1:20	B10824	13B8.2	TF, SA-βgal, T_8sen, CD8 Dextramers
CD4	KrOr	Beckman Coulter	1:20	A82789	13B8.2	T_8sen
CD4	Viobright R720	Miltenyi Biotec	1:50	REA623	130-127-378	T_8sen
CD4	PE-Cy™7	BD Biosciences	1:20	SK3	557852	CD4 Tetramers, Cytokines
CD19	AA700	Beckman Coulter	1:20	J3-1 19	B49212	CD4 Tetramers
CD45RA	PCy7	Beckman Coulter	1:20	2H4	B10821	TF, T_8sen
CD45RA	PercPVio770	Miltenyi Biotec	1:50	REA562	130-113-368	SA-βgal, T_8sen
CD45RA	PE-Vio770	Miltenyi Biotec	1:50	REA1047	130-117-746	T_8sen
CD56	PE-Vio615	Miltenyi Biotec	1:50	REA196	130-114-550	TF
CD56	APC	Miltenyi Biotec	1:50	REA196	130-113-310	SA-βgal, T_8sen
CD56	AA700	Beckman Coulter	1:20	N901	B92446	T_8sen
CD56	ECD	Beckman Coulter	1:20	N901	A82943	T_8sen
CD57	FITC	Miltenyi Biotec	1:50	TB03	130-122-935	TF, T_8sen, CD8 Dextramers
CD57	FITC	Beckman Coulter	1:10	NC1	IM0466U	T8sen
CD57	APC-Vio770	Miltenyi Biotec	1:50	REA769	130-111-813	CD4 Tetramers, Cytokines
CD57	PE-Vio770	Miltenyi Biotec	1:50	REA769	130-111-812	SA-βgal, T_8sen
CD8	VioGreen	Miltenyi Biotec	1:50	REA734	130-110-684	TF, SA-βgal, T_8sen, CD8 Dextramers, CD4 Tetramers, Cytokines
CD8	APC	Beckman Coulter	1:20	IM2469	B9.11	T_8sen
Eomes	PE	BD Biosciences	1:10	X4-83	566749	TF
IFN-γ	FITC	Miltenyi	1:50	REA600	130-113-497	Cytokines
KLRG1	BV421™	Biolegend	1:20	2F1	138414	TF, SA-βgal, T_8sen
KLRG1	PE	Miltenyi Biotec	1:10	REA261	130-103-638	T_8sen
KLRG1	Vioblue	Miltenyi Biotec	1:50	REA261	130-123-526	T_8sen
KLRG1	PerCP-eFluor710	Invitrogen	1:20	13F12F2	46-9488-42	T_8sen
T-bet	PC5.5	Thermo/Invitrogen	1:10	4B10	45-5825-82	TF
TCR α/β	APC/Cy7	Biolegend	1:20	IP26	306728	T_8sen

Table S5. Plasma cytokine and soluble protein assays. The reference of the kits and sample dilutions are given for each soluble protein. LLOD: lower limit of detection

Target cytokine / soluble protein	Provider	Kit	Catalog number	Sample dilution	LLOD
IFN-β	MSD	U-PLEX Immuno-Oncology Group 1 (hu)	K151AEL-1	1:1	3.1 pg/ml
IL-6	MSD				0.33 pg/ml
IL-8	MSD				0.15 pg.ml
IL-10	MSD				0.14 pg/ml
IL-29/IFN-λ1	MSD				1.2 pg/ml
IP-10	MSD				0.49 pg/ml
PD-L1	MSD				0.09 pg/ml
TNF-α	MSD				0.51 pg/ml
IFN-α2a	MSD	S-PLEX Human IFN-α2a	K151P3S-1	1:1	4.9 fg/ml
VCAM-1	MSD	V-PLEX Human VCAM-1	K151SRD-1	1:1000	6 pg/ml
MPO	MSD	R-PLEX Human MPO	K1514ER-2	1:20	11 pg/ml
Elastase	Abcam	Human Neutrophil Elastase ELISA Kit	ab270204	1:2000-1:10000	47 pg/ml

Table S6. TaqMan^{TM} assays references used for RT-qPCR

Gene name	Translated protein	TaqMan^{TM} probes references	Provider
MX1	Mx1	Hs00895608_m1	ThermoFisher Scientific
IFITM1	IFITM1	Hs00705137_s1	
IFIT1	IFIT1	Hs01675197_m1	
IFI44	IFI44	Hs00197427_m1	
LY6E	Ly6E	Hs00158942_m1	
ACTB	Actin beta	Hs99999903_m1	
